# β-arrestin1 regulates astrocytic reactivity via Drp1-dependent mitochondrial fission: implications in postoperative delirium

**DOI:** 10.1186/s12974-023-02794-x

**Published:** 2023-05-11

**Authors:** Fuzhou Hua, Hong Zhu, Wen Yu, Qingcui Zheng, Lieliang Zhang, Weidong Liang, Yue Lin, Fan Xiao, Pengcheng Yi, Yanhong Xiong, Yao Dong, Hua Li, Lanran Fang, Hailin Liu, Jun Ying, Xifeng Wang

**Affiliations:** 1 Department of Anesthesiology, The Second Affiliated Hospital of Nanchang University, 1# Minde Road, Nanchang, 330006 Jiangxi People’s Republic of China; 2 Key Laboratory of Anesthesiology of Jiangxi Province, 1# Minde Road, Nanchang, 330006 Jiangxi People’s Republic of China; 3 Department of Neurosurgery, The Second Affiliated Hospital of Nanchang University, 330006 Nanchang, Jiangxi People’s Republic of China; 4 Department of Anesthesiology, The First Affiliated Hospital of Gannan Medical University, Ganzhou, 341000 Jiangxi People’s Republic of China; 5 Department of Anesthesiology, First People’s Hospital of Yihuang County, Fuzhou, 344400 Jiangxi People’s Republic of China; 6 Department of Statistics, Jiangxi University of Finance and Economics, Nanchang, 330013 Jiangxi People’s Republic of China; 7 Department of Anesthesiology, The First Affiliated Hospital of Nanchang University, 17# Yong Wai Zheng Street, Nanchang, 330006 Jiangxi People’s Republic of China

**Keywords:** Postoperative delirium, Neuroinflammation, Astrocytes, β-Arrestins, Mitochondrial fission

## Abstract

Postoperative delirium (POD) is a frequent and debilitating complication, especially amongst high risk procedures, such as orthopedic surgery. This kind of neurocognitive disorder negatively affects cognitive domains, such as memory, awareness, attention, and concentration after surgery; however, its pathophysiology remains unknown. Multiple lines of evidence supporting the occurrence of inflammatory events have come forward from studies in human patients’ brain and bio-fluids (CSF and serum), as well as in animal models for POD. β-arrestins are downstream molecules of guanine nucleotide-binding protein (G protein)-coupled receptors (GPCRs). As versatile proteins, they regulate numerous pathophysiological processes of inflammatory diseases by scaffolding with inflammation-linked partners. Here we report that β-arrestin1, one type of β-arrestins, decreases significantly in the reactive astrocytes of a mouse model for POD. Using β-arrestin1 knockout (KO) mice, we find aggravating effect of β-arrestin1 deficiency on the cognitive dysfunctions and inflammatory phenotype of astrocytes in POD model mice. We conduct the in vitro experiments to investigate the regulatory roles of β-arrestin1 and demonstrate that β-arrestin1 in astrocytes interacts with the dynamin-related protein 1 (Drp1) to regulate mitochondrial fusion/fission process. β-arrestin1 deletion cancels the combination of β-arrestin1 and cellular Drp1, thus promoting the translocation of Drp1 to mitochondrial membrane to provoke the mitochondrial fragments and the subsequent mitochondrial malfunctions. Using β-arrestin1-biased agonist, cognitive dysfunctions of POD mice and pathogenic activation of astrocytes in the POD-linked brain region are reduced. We, therefore, conclude that β-arrestin1 is a promising target for the understanding of POD pathology and development of POD therapeutics.

## Introduction

Postoperative delirium (POD) is a neuropsychiatric complication occurring after high risk procedures with general anesthesia, such as orthopedic surgery [1]. It is mainly characterized as neurocognitive disorders such as memory loss, attention deficits, and concentration failure in vulnerable patients such as the elderly [2, 3]. Despite its acute course, typically 1–3 days after surgery plus anesthesia, delirium has devastating consequences including increased postoperative mortality, decreased quality of life and long-term risk for Alzheimer’s disease (AD) [2, 4]. However, the etiology of POD remains to be determined, for the lack of animal models to study the neuropathogenesis and targeted interventions. Orthopedic surgery is performed routinely, especially in older adults, to repair common bone injuries. As many as 50% of patients suffer from delirium and cognitive dysfunctions after orthopedic surgery; therefore, researchers have developed a mouse model of tibial fracture to study the impacts of orthopedic surgery on the central nervous system (CNS) and investigate the pathogenesis of POD [5, 6]. Studies in human patients’ brain and bio-fluids (CSF and serum), as well as in animal models for POD support the occurrence of inflammatory events as a critical driver of POD [7–9]. In line with these concepts, our previous studies have proved that treatments targeting inflammatory responses in POD presents promising therapeutic potentials [10].

Microglia and astrocytes are key regulators of inflammatory responses in the CNS. Glial activation appears to be a double-edged sword in neural diseases and is traditionally categorized as neurotoxic (M1 microglia and A1 astrocytes) or neuroprotective (M2 microglia and A2 astrocytes) [11, 12]. Meanwhile, the phenotypic distribution of glia may vary in different pathological conditions [11, 12]. Previous studies have verified that immune episodes after surgery trigger the release of pro-inflammatory cytokines from microglia, including tumor necrosis factor-α (TNF-α) and interleukin-β (IL-β), which correlates to more severe forms of dementia and AD pathogenesis [13–15]. Surgery-induced activation of complement component 3/complement component 3 receptor (C3/C3R) signaling pathway, which is later regarded as the most characteristic marker of neurotoxic astrocytes, is reported to worsen cognitive impairments in perioperative neurocognitive disorders [16]. These researches collectively infer the toxic/pathogenic glial activation in the progression of POD.

Guanine nucleotide-binding protein (G protein)-coupled receptors (GPCRs) constitute the largest known super family for signal transduction and regulate fundamental physiological functions essential to life, such as metabolism, neurotransmission, immune responses, and homeostasis. For their multiple functions, they are also the most intensively studied drug targets [17]. Conformational changes of the receptor increase their affinity for the multifunctional GPCR regulatory or adaptor proteins known as the β-arrestins, including two super family members β-arrestin1 and β-arrestin2 [18]. The association between receptors and β-arrestins blocks subsequent G protein activation and has an important role in traditional GPCR desensitization. In addition to their canonical role in binding to phosphorylated GPCRs to evoke receptor desensitization and endocytosis [18], β-arrestins have much broader effects including cellular signaling transduction and epigenetic modifications by interacting with numerous non-receptor binding partners [19, 20]. For example, β-arrestins-associated complexes combine with extracellular signal-regulated kinase (ERK) to mediate ERK signaling pathways, as are the Raf-1, MEK1, and ubiquitin dependent signaling [20–22]. Besides, β-arrestins can also act as a dynamic nuclear linker of several transcriptional factors and co-factors, such as β-catenin, NF-ĸB and the hypoxia-inducible factor 1α (HIF-1α), mediating the epigenetic regulation of genes associated with pathological progression [23]. Studies using β-arrestins knockout (KO) mice have shown that β-arrestins regulate numerous physiologic and pathophysiologic processes, highlighting their potential roles as therapeutic targets. For example, β-arrestin2 is found to regulate NLRP3 inflammasome complex formation by interacting with NLRP3 protein, thus involving in the pathogenesis of Parkinson's disease (PD) [24]. Furthermore, β-arrestin2-biased agonist prevents dopaminergic neuron degeneration [25]. Another study demonstrates that β-arrestin1 KO mice diminishes amyloid-β pathology by regulating γ-secretase complex assembly [26]. Although there are so many interesting studies emphasizing their important roles, whether G protein-coupled receptors and the signaling molecules may be promising therapeutic targets of POD are unknown.

With this study, we showed that β-arrestin1 decreased in the tibial fracture mouse model for POD, accompanying the pro-inflammatory micro-environment in the hippocampus of POD model mice, while β-arrestin2 showed no change. This decreased expression of β-arrestin1 was mainly found in the reactive astrocytes. Using β-arrestin1 KO mice, we found that β-arrestin1 deficiency aggravated pathological phenotypes and behavioral abnormalities of POD mice. Further investigations in vitro demonstrated the β-arrestin1 interacted with dynamin-related protein 1 (Drp1) to inhibit excessive mitochondrial fragmentation, which was mediated by mitochondrial translocation of Drp1. Furthermore, activation of β-arrestin1 signals by β-arrestin1-biased agonist Carvedilol (Carv) protected POD mice from cognitive dysfunctions as well as neurotoxic astrocytic activation. Our study expands the pathological mechanism of postoperative delirium and provides a rationale for clinical medication.

## Materials and methods

### Animals

C57BL/6J mice (3 months) were obtained from Medical Animal Experiment Center of Nanchang University. β-Arrestin1 knockout mice were obtained from Jackson’s laboratory (stock number: 011131). Mice were bred and maintained in the Animal Resource Centre of Nanchang University. All animal experimental protocols were approved by the Institutional Animal Care and Use Committee of Nanchang University and were performed in accordance with the standards established by the guideline of Nanchang University.

## Surgical model

Tibial fracture was performed as previously described [6]. Briefly, mice were randomly divided into groups needed and then anesthetized with 1.8% isoflurane and oxygen at 2 L/min in the small animal anesthesia machine (RWD Life Science). Muscles were disassociated following an incision on the left hindpaw. A 0.38-mm stainless steel pin was inserted into the tibia intramedullary canal, followed by osteotomy, and the incision was sutured. 2% lidocaine solution was applied locally before the incision, and 1% tetracaine hydrochloride mucilage was applied to the wound twice daily to treat the pain.

For Carv administration, 3.2 mg/kg Carvedilol and vehicle control were delivered every day to mice in drinking water for 30 days [27]. And then, the mice were established the tibial fracture mouse model for POD, during which Carv was administered continuously until behavioral test.

### Behavioral tests

#### Y-maze test

Y-maze was performed referring to our previous study [10]. The Y-maze apparatus consisted of three arms separated by an angle of 120°. In the Y-maze tests, all mice were applied two trials separated by an interval of 1 h. In the first trial, each mouse was placed at the center of a symmetrical Y-maze and was allowed to explore freely through the maze, except for the novel arm, for 5 min. In the second trial, all experimental arms opened and mice were placed in the maze in the starting arm, with free access to all three arms. Bouts of novel arm entry and duration in the novel arm were recorded. The hind-paw of a mouse entering one arm was defined as one arm entry. The total time of the experiment was 5 min.

#### Morris water maze

Morris water maze test was conducted in a circular tank (1.1 m in diameter) containing opaque water (22 ± 1 °C) and a platform (10 cm in diameter) submerged 1.0 cm under the water. The water tank was dimly lit and surrounded by a white curtain. The maze was virtually divided into four quadrants, with one containing the platform (diameter 10 cm). Four prominent cues were placed outside the maze as spatial references. Mice were placed in the water facing the tank wall at different start positions across trials in a quasi-random fashion to prevent strategy learning. Mice were allowed to search for the platform for a duration of 1 min; if the mice did not find the platform during 1 min, they were guided towards the platform and then required to stay for 20 s. Each mouse went through four trials (one trial from each quadrant) per day for five consecutive days. After each trial, the mouse was dried and placed back into its cage until the start of the next trial. All mouse movements were recorded via a video tracking system, which calculated distances moved and time required to reach the platform (latency). The spatial probe trial was conducted 24 h after the last training session (on day 6). For the probe trial, the platform was removed and mice were allowed to swim for 1 min. Time to reach the invisible platform on the probe trial day were was recorded and calculated. Times of mice crossing the hidden platform were recorded and calculated.

### Primary cell cultures and treatments

For primary astrocyte culture, the brain tissues of WT and β-arrestin1^−/−^ neonatal mice aged 1–3 d were stripped of meninges and blood vessels under a microscope. Then, the tissues were digested with 0.25% trypsin (Gibco, #27250018) for 2 min and terminated by Dulbecco’s modified Eagle’s medium (DMEM, Gibco, #12100-046) supplemented with 10% fetal bovine serum (FBS, Gibco, #10437028). Cell suspension was filtered with a 40 μm filter (BD falcon, #352340) and centrifuged at 1000*g* for 5 min. Cells were re-suspended in DMEM supplemented with 10% FBS and 1% penicillin/streptomycin (P/S, Gibco, #15640055) and then plated in culture dishes (Corning, #430167). The culture medium was replaced with fresh medium 24 h later and then refreshed every 3 days. After the cells grew to 90% on the 7th–9th day, the cells were split into culture plates as needed.

### Immunohistochemical (IHC) analysis

The brain tissues were dehydrated with 20% sucrose dissolved in phosphate-buffered saline (PBS) and then 30% sucrose-PBS for 3 days, respectively, after post-fixed in 4% paraformaldehyde (PFA). Then, the brains were cut into 25 μm-thick slices. For immunohistochemical analysis, brain sections are incubated with 3% hydrogen peroxide to quench the endogenous peroxidase activity before blocking with 5% BSA/PBST. After overnight incubation with primary antibody in 4 ℃, the HRP-labeled secondary antibody was incubated at room temperature for 1 h, and then the slices were rinsed with PBS for three times. Finally, the slices were visualized by the Diaminobenzidin (DAB, Boster, #AR1002) reaction for 5 min. Stereo Investigator software was used to image and count the number of positive cells under the microscope (Olympus).

The primary antibodies used for immunohistochemical staining were as follows: mouse anti-GFAP antibody (1:300, Cell Signaling Technology, #3670), rabbit anti-Iba-1 antibody (1:500, Wako, #019-19741).

### Immunofluorescent analysis

For immunofluorescence of brain slices, the sections were blocked with 5% Fetal Bovine Serum in PBST (0.3% Triton X-100) for 1 h, followed by overnight incubation with primary antibody in 4 ℃. And then, the sections were washed with PBS and incubated with Alexa Fluor® 488 goat anti-rabbit IgG antibody (1:1000, Invitrogen, #A11008), Alexa Fluor® 488 goat anti-mouse IgG antibody (1:1000, Invitrogen, #A11001), Alexa Fluor® 555 goat anti-mouse IgG antibody (1:1000, Invitrogen, #A21422) or Alexa Fluor® 555 goat anti-rabbit IgG antibody (1:1000, Invitrogen, #A21428) for 1 h at room temperature. The sections were rinsed with PBS and then mounted onto adhesive slides. Nucleus were stained with Hoechst 33,342 (1 μl diluted in 1000 μl PBS) for 15 min. Fluorescently labeled sections were visualized with an Olympus scanning microscope. For immunocytochemical staining, primary cells were rinsed with 0.1 M PBS and fixed with 4% PFA for 20 min. Cell cultures on the cell slides were then followed the same procedures as immunofluorescence of brain slices. The primary antibodies used for immunofluorescent staining were as follows: rabbit anti-β-arrestin1 (1:200, Cell Signaling Technologies, #12697), mouse anti-β-arrestin1 (1:200, Santa Cruz, #sc-53780), mouse anti-GFAP antibody (1:300, Cell Signaling Technology, #3670), rabbit anti-Iba-1 antibody (1:500, Wako, #019-19741), rabbit anti-C3 antibody (1:200, abcam, #ab11887), rabbit anti-Serping1 antibody (1:300, proteintech, #12259-1-AP), mouse anti-Drp1 (1:300, Santa Cruz, #sc-101270), rabbit anti-TOMM20 (1:300, Proteintech, #11802-1-AP).

### Western blotting analysis

Mouse brain tissues and cell culture extract lysates were quantified by QuantiPro™ BCA Assay Kit (abcam, #ab102536). 30 μg proteins were separated by sodium dodecyl sulfate polyacrylamide gel electrophoresis (SDS-PAGE) and then electrophoretically transferred to polyvinylidene difluoride (PVDF) membranes (Millipore, #IPVH00010). After blocking with 10% nonfat dry milk in Tris-buffered saline (20 mM Tris–HCl, 500 mM NaCl, pH 7.4) with Tween 20 (Aladdin, #T104863), the membranes were then probed with the following primary antibodies overnight at 4 °C: mouse anti-GFAP antibody (1:1000, Cell Signaling Technology, #3670), rabbit anti-Iba-1 antibody (1:1000, Wako, #019-19741), rabbit anti-β-arrestin1 (1:1000, Cell Signaling Technologies, #12697), rabbit anti-β-arrestin2 (1:200, Cell Signaling Technologies, #3857), rabbit anti-C3 antibody (1:1000, abcam, #ab11887), rabbit anti-Serping1 antibody (1:1000, proteintech, #12259-1-AP), rabbit anti-Psmb8 antibody (1:1000, proteintech, #14859-1-AP), mouse anti-Fis1 antibody (1:1000, Santa Cruz, #sc-376447), mouse anti-Drp1 antibody (1:1000, Santa Cruz, #sc-101270), rabbit anti-Drp1 antibody (1:1000, proteintech, #12957-1-AP), rabbit anti-COX IV antibody (1:1000, Cell Signaling Technology, #4850), mouse anti-β-actin antibody (1:3000, sigma, #a1978). The membranes were next incubated with a horseradish peroxidase-conjugated goat anti-mouse secondary antibody (1:5000, Thermo, #31430) or goat anti-rabbit secondary antibody (1:5000, Thermo, #31460) for 1 h. After washing, the membranes were scanned and analyzed using an Image Quant LAS 4000 Chemiluminescence (ECL) Imaging System (GE Healthcare, USA) by chemiluminescence western blotting detection reagents Pierce™ ECL (Thermo, #32132).

### Enzyme-linked immunosorbent assay (ELISA)

Hippocampal tissues were drawn from surgery-induced POD model mice after successful establishment of the mouse model. The brain tissue lysis was conducted the protein quantification assay to be homogenized to the same protein concentration. Brain IL-1β, TNF-α, IL-6, IL-4, IL-10, and BDNF were measured using ELISA kits for mouse according to the manufacturer’s instructions (ExCellBio).

### RNA isolation and quantitative real time PCR (RT-PCR)

Total RNA was extracted from hippocampal tissues and primary astrocytes using Trizol reagent (Invitrogen, #15596026) and reversely transcribed into cDNA using ChamQ Universal SYBR qPCR Master Mix (Vazyme, #Q711). Real-time PCR was performed in a 10 μl reaction system containing cDNA, primers, and HiScript III 1st Strand cDNA Synthesis Kit (Vazyme, #R312) with the ABI system. GAPDH was used as an internal control gene. qPCR primers were designed using a primer design tool, and their sequences were as follows:

**Table:** 

Gene	Forward primer	Reverse primer
il-1a	CGAAGACTACAGTTCTGCCATT	GACGTTTCAGAGGTTCTCAGAG
il-1b	TCAGGCAGGCAGTATCACTC	CATGAGTCACAGAGGATGGG
il-6	CCCCAATTTCCAATGCTCTCCT	CATAACGCACTAGGTTTGCCG
Tnf	CCCAC GTCGT AGCAA ACCA	GGCAG AGAGG AGGTT GACTT
C1qa	AAAGGCAATCCAGGCAATATCA	TGGTTCTGGTATGGACTCTCC
il-4	AGATGGATGTGCCAAACGTCCTCA	AATATGCGAAGCACCTTGGAAGCC
il-10	ATTTGAATTCCCTGGGTGAGAAG	CAGGGGAGAAATCGATGACA
il-13	TGAGACTCCGTTCTGGCCTC	CTCTTCATGCTTGGTACCCGAT
Mrc1	CTCTGTTCAGCTATTGGACGC	CGGAATTTCTGGGATTCAGCTTC
Arg1	CTCCAAGCCAAAGTCCTTAGAG	AGGAGCTGTCATTAGGGACATC
Egf	AGCAGCCCCTTCCCTAAGA	AGTGTGTCCGTCCTCCGAA
Vegf	CTGCCGTCCGATTGAGACC	CCCCTCCTTGTACCACTGTC
Bdnf	TCATACTTCGGTTGCATGAAGG	AGACCTCTCGAACCTGCCC
Gdnf	AAGTGGCACAGTTTTGCTGGA	GCTAACAGTGACATCACACAAGT
H2-T23	GGACCGCGAATGACATAGC	GCACCTCAGGGTGACTTCAT
Serping1	ACAGCCCCCTCTGAATTCTTT	GGATGCTCTCCAAGTTGCTC
H2-D1	TCCGAGATTGTAAAGCGTGAAGA	ACAGGGCAGTGCAGGGATAG
Ggta1	GTGAACAGCATGAGGGGTTT	GTTTTGTTGCCTCTGGGTGT
Ligp1	GGGGCAATAGCTCATTGGTA	ACCTCGAAGACATCCCCTTT
Gbp2	GGGGTCACTGTCTGACCACT	GGGAAACCTGGGATGAGATT
Fbln5	CTTCAGATGCAAGCAACAA	AGGCAGTGTCAGAGGCCTTA
Ugt1a	CCTATGGGTCACTTGCCACT	AAAACCATGTTGGGCATGAT
Fkbp5	TATGCTTATGGCTCGGCTGG	CAGCCTTCCAGGTGGACTTT
Psmb8	CAGTCCTGAAGAGGCCTACG	CACTTTCACCCAACCGTCTT
Srgn	GCAAGGTTATCCTGCTCGGA	TGGGAGGGCCGATGTTATTG
Amigo2	GAGGCGACCATAATGTCGTT	GCATCCAACAGTCCGATTCT
C3	CCAGCTCCCCATTAGCTCTG	GCACTTGCCTCTTTAGGAAGTC
Clcf1	CTTCAATCCTCCTCGACTGG	TACGTCGGAGTTCAGCTGTG
Ptx3	AACAAGCTCTGTTGCCCATT	TCCCAAATGGAACATTGGAT
S100a10	CCTCTGGCTGTGGACAAAAT	CTGCTCACAAGAAGCAGTGG
Sphk1	GATGCATGAGGTGGTGAATG	TGCTCGTACCCAGCATAGTG
Cd109	CACAGTCGGGAGCCCTAAAG	GCAGCGATTTCGATGTCCAC
Ptgs2	GCTGTACAAGCAGTGGCAAA	CCCCAAAGATAGCATCTGGA
Emp1	GAGACACTGGCCAGAAAAGC	GCAGCGATTTCGATGTCCAC
Slc10a6	GCTTCGGTGGTATGATGCTT	CCACAGGCTTTTCTGGTGAT
Tm4sf1	GCCCAAGCATATTGTGGAGT	AGGGTAGGATGTGGCACAAG
B3gnt5	CGTGGGGCAATGAGAACTAT	CCCAGCTGAACTGAAGAAGG
Cd14	GGACTGATCTCAGCCCTCTG	GCTTCAGCCCAGTGAAAGAC
Ccr10	GGACTTTACTCCGGGTACGAT	CAGGGAGACACTGGGTTGGA
Arrb2	GGCAAGCGCGACTTTGTAG	GTGAGGGTCACGAACACTTTC
Rgs1	TCTGGGATGAAATCGGCCAAG	GCATCTGAATGCACAAATGCTT
Tdrd1	ATGATGCCACGGAATAATTTGGA	GGTGGGGTGGTTATGCTGT
Lrp1	ACTATGGATGCCCCTAAAACTTG	GCAATCTCTTTCACCGTCACA
Cxcl5	TCCAGCTCGCCATTCATGC	TTGCGGCTATGACTGAGGAAG
Prad3	GGAGATGGCCGCATGAAAGTT	CTCCAAGCGATGCACCTGTAT
Rgd10	TCCATGACGGAGATGGGAG	AACAAGACATTCTCTTCGCTGAA
Lrp6	TTGTTGCTTTATGCAAACAGACG	GTTCGTTTAATGGCTTCTTCGC
Calcrl	ATCTCAGCAGAGTCGGAAGAA	CAGGTCCTATTGCAGTAAAGGC
Arrb1	AAGGGACACGAGTGTTCAAGA	CCCGCTTTCCCAGGTAGAC
P2rx7	GACAAACAAAGTCACCCGGAT	CGCTCACCAAAGCAAAGCTAAT
Adgrf5	GGGTTTCGGTCTTGCCACA	CTTCCTGCACCTTCTGATCCC
Ptger4	ACCATTCCTAGATCGAACCGT	CACCACCCCGAAGATGAACAT
Gpr162	CTCCCTACGCTCAAACGCATT	CCGCCATGAGTATATGAGTACCG
gapdh	TGTAGACCATGTAGTTGAGGTCA	AGGTCGGTGTGAACGGATTTG

### Detection of mitochondrial functions by fluorescent dyes

#### MitoSOX fluorescent dye

MitoSOX (Invitrogen, #M36008) is a fluorescent dye which can be oxidized by mitochondrial superoxide of live cells and exhibit red fluorescence. This fluorescent dye is used for the detection of reactive oxygen species (ROS). After treatment, the culture medium of primary astrocytes was sucked off and the cells were stained with 5 μM MitoSOX at 37 ℃ in dark for 10 min. The cells were then rinsed twice and then resuspended with cold PBS containing 1% FBS for flow cytometric analysis (FACScan; Becton Dickinson).

#### JC-1 fluorescent dye

JC-1 fluorescence probe (Invitrogen, #T3168) was a membrane permeable dye to determine mitochondrial membrane potential. Upon membrane polarization, JC-1 was transformed from mitochondrial aggregates with emission of a strong red fluorescence (Ex = 585 nm, Em = 590 nm) to cytoplasm monomers in green fluorescence (Ex = 514 nm, Em = 529 nm). After the treatment, the culture medium of astrocytes was discarded, and the cells were added with fresh JC-1 solution (final concentration is 10 μg/ml) and incubated at 37 ℃ for 30 min. After rinsing with PBS for three times, the cells were digested and re-suspended in cold PBS containing 1% FBS for flow cytometric analysis (FACScan; Becton Dickinson).

#### Mitotracker green

Mitotracker green (Beyotime, #C1048) was used to stain the mitochondria. After the treatment, astrocytic culture medium was discarded and the cells were added with fresh Mitotracker green solution and incubated at 37 ℃ for 40 min. After rinsing with PBS for three times, the cells were imaged using a fluorescence microscope.

### Determination of ATP content

The ATP content in primary astrocytes was detected using the enhanced ATP assay kit (Beyotime, #S0027) in accordance with the manufacturer protocol. Cells were lysed in the ATP lysis buffer, and the cell precipitates were lysed and then homogenized with the ATP lysis buffer. ATP contents were determined using a luminometer.

### Co-immunoprecipitation (CO-IP) assay

Proteins from primary astrocytes were lysed in cell lysis buffer after experimental treatments. Lysates were incubated with the anti-β-arrestin1 (Cell Signaling Technologies, #12697), anti-Drp1 antibody (proteintech, #12957-1-AP), anti-Drp1 antibody (Santa Cruz, #sc-101270), anti-Fis1 antibody (Santa Cruz, #sc-376447), or mouse IgG (Cell Signaling Technology, #3420) or rabbit IgG (Cell Signaling Technology, #3423) overnight, followed by incubation with protein A/G-agarose beads (Santa Cruz, #sc-2003) for another 4 h in 4 ℃. After centrifugation at 500×*g* for 3 min, the pellet was washed with pre‐cooled PBS, and the beads were boiled in loading buffer for 5 min. Then, the supernatants were collected and subject to western blot analysis for β-arrestin1, Drp1 and Fis1.

### Mitochondrial protein extraction

Mitochondria of primary astrocytes were extracted using Cell Mitochondria Isolation Kit for Cultured Cells (abcam, #ab110170). Primary astrocytes were digested by trypsin and then centrifuged to collect the pellets. After suspending cells in reagent A and incubating for 10 min, the cells were homogenized using dounce homogenizer. Then, homogenates were centrifuged at 1000*g* for 10 min at 4 ℃ and retain supernatant. The supernatants were then proceeding to centrifuge at 12,000*g* for 15 min at 4 ℃ and retain pellet. After centrifugation, the pellets were re-suspended in reagent C and analyzed for the protein concentration of the mitochondrial part, while the supernatants were centrifuged at 12,000*g* for 10 min at 4 ℃ and then collected the supernatants of this step, which were cytoplasmic protein without mitochondria.

### Oxygen consumption rates (OCR) analysis 

Mitochondrial respiratory functions of primary astrocytes were measured oxygen consumption rate (OCR) using Seahorse xF96. Primary astrocytes were plated on Seahorse xF96 plates and challenged with untreated microglia culture medium (MCM) and LPS-incubated MCM. Three cycles of baseline measurement of OCR were taken followed by 3 cycles of sequential measurements after injection of oligomycin (ATP synthase inhibitor), carbonyl cyanide-4-(trifluoromethoxy) phenylhydrazone (FCCP, mitochondrial respiration uncoupler), and antimycin A (Complex III inhibitor) in conjunction with rotenone (Complex I inhibitor). OCR data was normalized to cell numbers.

### Stereotactic injection of AAV

For astrocyte-specific β-arrestin1 knockdown, adeno-associated virus (AAV) with β-arrestin1 siRNA and vectors containing a astrocytic GFAP promoter (AAV9-GFAP-mβ-arrestin1-GFP) was packaged. In addition, the AAV-GFP vector without β-arrestin1 siRNA was used as a negative control AAV (NC AAV). AAV-GFAP-mβ-arrestin1-GFP vector (siβ-arrestin1 AAV) and the NC AAV (titers > 1.0 × 10^13^) were delivered by bilateral stereotactic injections into the dorsal hippocampus (AP: − 2.00; ML: 1.50; DV: − 1.80) and ventral hippocampus (AP: − 2.80; ML: 3.00; DV: − 4.00). The mice were allowed to recover from the virus injection for 2 weeks before anesthesia/surgery. Successful injection of virus was confirmed by fluorescence.

### Statistical analyses

All data are represented as mean ± SEM. with at least 3 independent experiments. Statistical analyses were performed using GraphPad Prism 7.0. Student’s unpaired two-tailed t-test, one-way ANOVA or two-way ANOVA was conducted according to test requirements. Difference was considered significant at *P* < 0.05. The number of replicates and repeats of individual experiments and statistical tests are indicated in the legends.

## Results

### Orthopedic surgery induces glial activation and pro-inflammatory phenotypes in the hippocampus

**Fig. 1:**
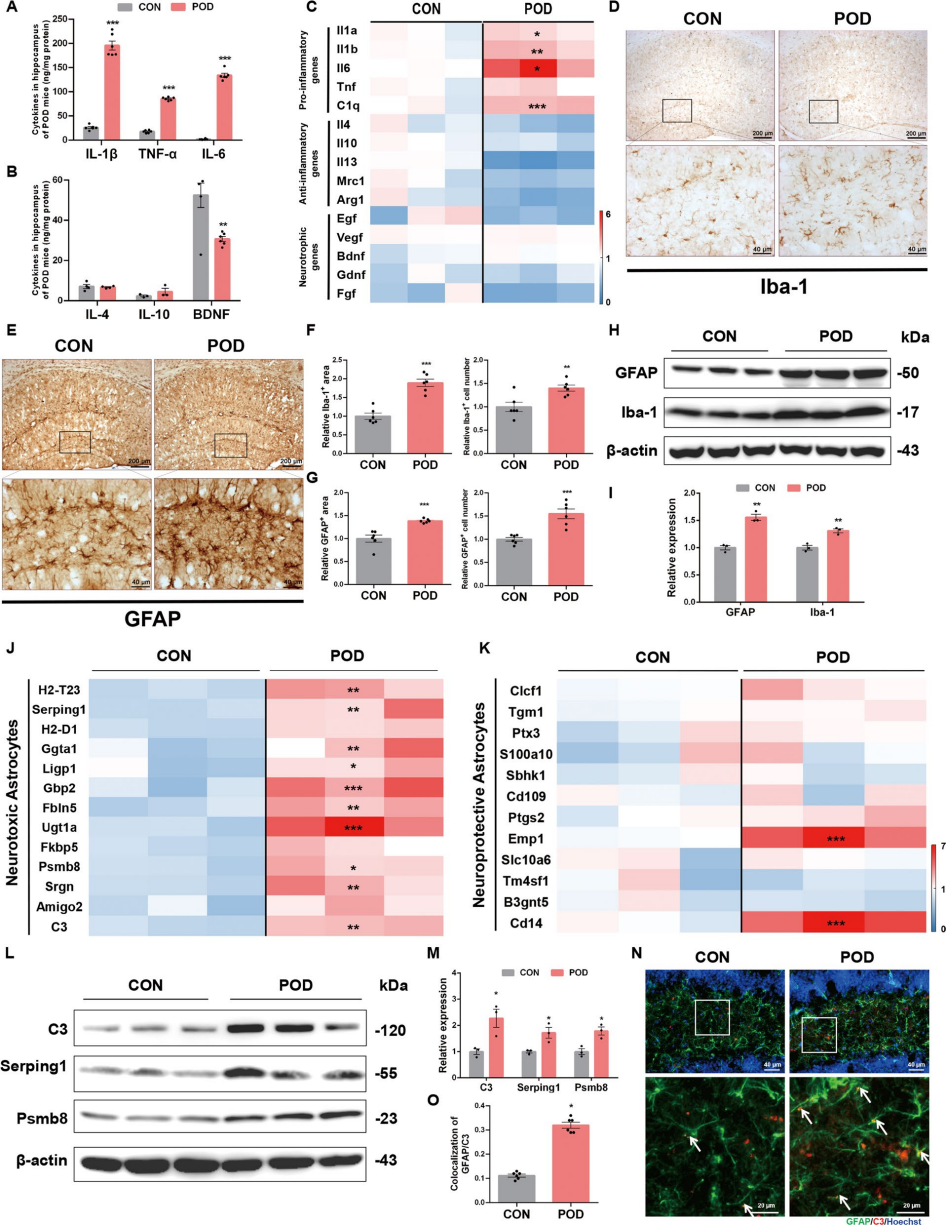
Orthopedic surgery induces glial activation and pro-inflammatory phenotypes in the hippocampus.** A** Mice were established orthopedic surgery-induced POD model. Levels of pro-inflammatory cytokines in the hippocampus were analyzed by ELISA. **B** Levels of anti-inflammatory and neurotrophic cytokines in the hippocampus were analyzed by ELISA. **C** mRNA levels of pro-inflammatory and anti-inflammatory genes as well as neurotrophic genes in the hippocampus were analyzed by RT-PCR. **D** Immunohistochemical staining of Iba-1 in the hippocampus of CON and POD mice. **E** Immunohistochemical staining of GFAP in the hippocampus of CON and POD mice. **F** Analysis of Iba-1-positive area and cell numbers in the hippocampus. **G** Analysis of GFAP-positive area and cell numbers in the hippocampus. **H** Expression of GFAP and Iba-1 in the hippocampus of CON and POD mice. **I** Densitometric analysis of GFAP and Iba-1. **J** Heatmap of neurotoxic astrocytes transcripts in the hippocampus of CON and POD mice. **K** Heatmap of neuroprotective astrocytes transcripts in the hippocampus of CON and POD mice. **L** Expression of C3, Serping1 and Psmb8 in the hippocampus of CON and POD mice. **M** Densitometric analysis of C3, Serping1 and Psmb8. **N** Double immunofluorescent staining of astrocytic pan-active marker GFAP and neurotoxic astrocytic marker C3 in hippocampus of CON and POD mice. **O** Relative co-localized signals of the GFAP-positive and C3-positive immunofluorescent particles between CON and POD group. Data are analyzed by unpaired Student’s t-test. ^*^P < 0.05, ^**^P < 0.01 and ^***^P < 0.001 vs*.* the CON group. Values are presented as means ± SEM from at least three independent experiments

Neuroinflammation is an intertwined consequence of glial activation, inflammatory cytokines release and indirect effects of non-inflammatory events [9]. To figure out the interrelationships between multiple cellular functions and POD pathology, mainly the effect of neuroinflammation on the disease trajectory of POD, we assessed the hippocampal micro-environment of mice after orthopedic surgery. The release of pro- and anti-inflammatory cytokines in POD mouse model is the most predominant phenotypes. As shown in Fig. 1A, orthopedic surgery induced significantly increased levels of pro-inflammatory cytokines, including IL-1β, TNF-α and IL-6 in the hippocampal homogenates, while no marked changes were observed in anti-inflammatory cytokines including IL-4 and IL-10 levels (Fig. 1B). Emerging findings suggest that neurotrophic factors may also affect the functionality of the neuroinflammation and is associated with postoperative delirium [28, 29]. Brain derived neurotrophic factor (BDNF), one type of neurotrophic factors decreased markedly (Fig. 1B). We next compared the mRNA levels of these inflammatory and neurotrophic genes in the hippocampus of control mice and POD mice. Pro-inflammatory genes including Il-1a, Il-1b, Il6 and C1q were significantly higher than that of control group, while anti-inflammatory and neurotrophic genes showed decreased trends or no significant change (Fig. 1C). These results implied POD induced a pro-inflammatory micro-environment in the hippocampus.

As microglia and astrocytes are the active players of brain inflammatory events and mediates the release of neurotrophic factors [30], we, therefore, used immunohistochemical method to detect the activated states of microglia and astrocytes in hippocampal tissues. After surgery, activation of microglia and astrocytes, manifested as increased numbers and enlarged areas in the immunohistochemical staining of glial fibrillary acidic protein (GFAP) and ionized calcium-binding adapter molecule 1 (Iba-1), were shown in dentate gyrus (DG) of mice (Fig. 1D–G). Immunoblotting analysis of these specific markers consistently showed that surgery induced the increased expression of Iba-1 and GFAP (Fig. 1H, I). Glial activation may be beneficial (promotion of tissue repair by increasing anti-inflammatory cytokines and neurotrophic factors) or detrimental (exacerbation of tissue damage by increasing pro-inflammatory genes and neurotoxic molecules) [31]. Previous studies have verified that pro-inflammatory mediators (IL-1, TNF-α, etc.) are sufficient to activate neurotoxic reactive astrocytes, termed the neurotoxic astrocytes [32]. Here we demonstrated that the astrocytic activation in POD mouse model was skewed towards the neurotoxic reactivity, as we found that neurotoxic astrocytes markers synchronously increased in the hippocampus of the surgery-treated mice compared with that of control group mice (Fig. 1J), while markers of protective astrocytes changed inconsistently with some of them increased and some unchanged (Fig. 1K). And also, protein levels of some characteristic markers of neurotoxic astrocytes were up-regulated significantly in the POD group (Fig. 1L, M). Co-localization of C3, the most characteristic marker, and GFAP was significantly increased after orthopedic surgery (Fig. 1N, O). These results suggest that orthopedic surgery induces injurious inflammatory responses in the hippocampus.

### β-arrestin1 is decreased in the reactive astrocytes of orthopedic surgery-treated mice

**Fig. 2:**
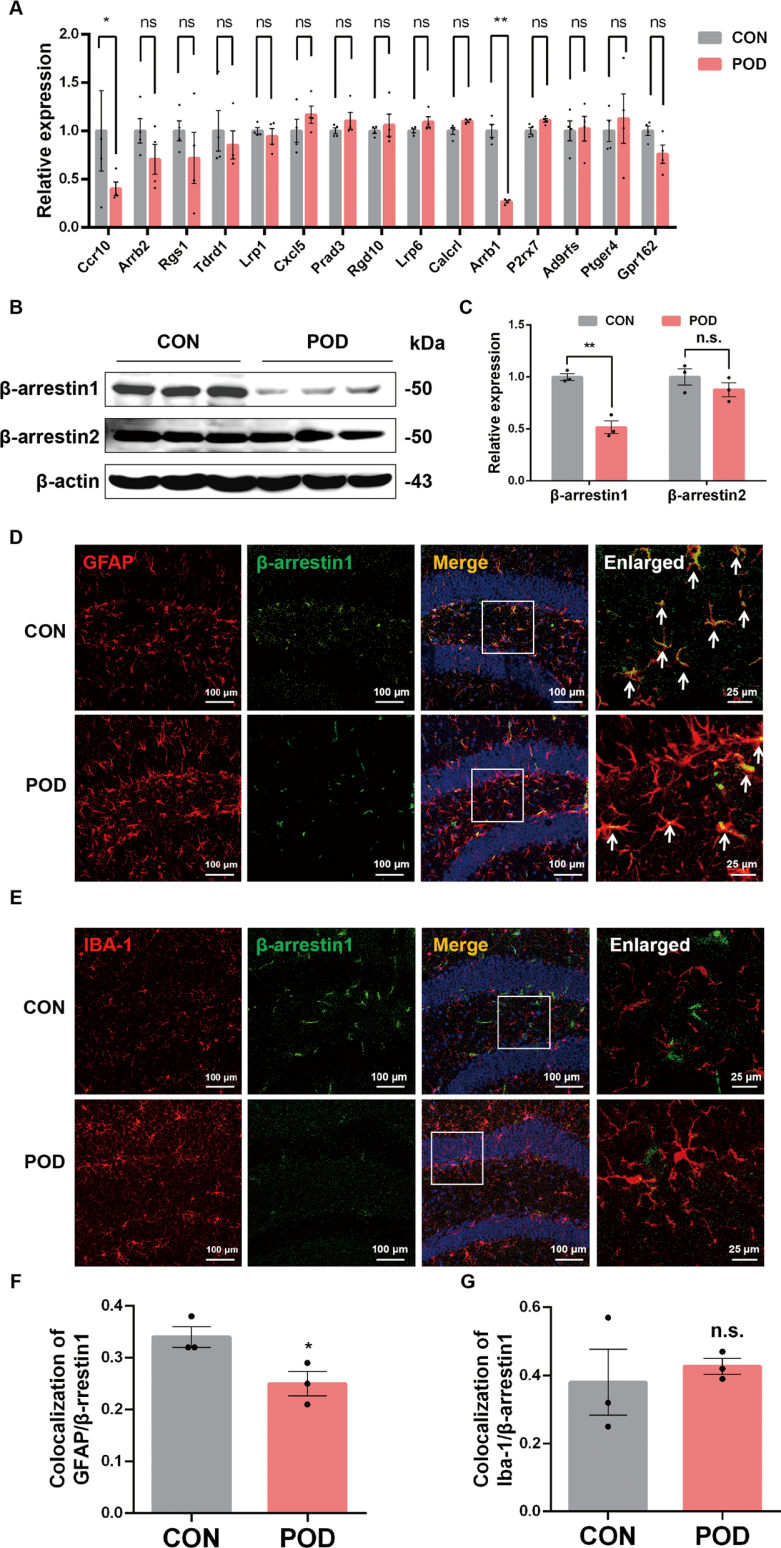
β-arrestin1 is decreased in the reactive astrocytes of orthopedic surgery-treated mice.** A** mRNA levels of GPCR-related genes in the hippocampus were analyzed by RT-PCR. **B** Expression of β-arrestin1 and β-arrestin2 in the hippocampus of CON and POD mice. **C** Densitometric analysis of β-arrestin1 and β-arrestin2. **D** Representative immunofluorescent staining of GFAP (red) and β-arrestin1 (green) in hippocampal slices of CON and POD mice. **E** Representative immunofluorescent staining of Iba-1 (red) and β-arrestin1 (green) in hippocampal slices of CON and POD mice. **F** Relative co-localized signals of the GFAP-positive and β-arrestin1-positive immunofluorescent particles between CON and POD group. **G** Relative co-localized signals of the Iba-1-positive and β-arrestin1-positive immunofluorescent particles between CON and POD group. Data are analyzed by unpaired Student’s t-test. n.s means no significance. ^*^P < 0.05 and ^**^P < 0.01 vs. the CON group. Values are presented as means ± SEM from at least three independent experiments

GPCRs are the most intensively studied drug targets for their substantial involvement in human pathophysiology and their pharmacological tractability [17]. To analyse the association of GPCR family members with the inflammatory pathogenesis of POD, we performed a gene assay to screen the expression of GPCR family genes in the hippocampus of POD mice. We demonstrated that Ccr10 and β-arrestin1 were significantly decreased in the POD mice brain compared with the control mice, among which the β-arrestin1 mRNA levels lowered most remarkably (Fig. 2A). Combining these RT-PCR results with the previous study, which implicated that β-arrestins as the multifunctional proteins downstream of GPCRs, are involved in the cognitive impairment and dementia of the amyloid-β pathology [26], we, therefore, investigated the role of β-arrestins in orthopedic surgery-induced postoperative delirium. We supplementarily compared the protein levels of β-arrestin1 and β-arrestin2 in samples from the hippocampus of POD mice with control mice. Consistent with the RT-PCR results, β-arrestin1 were significantly decreased, whereas levels of β-arrestin2 showed no significant change in the hippocampal tissues of POD mice (Fig. 2B, C). Next, we investigated the cellular distribution of decreased β-arrestin1 in the hippocampus focusing on the two main inflammatory cell types in the CNS: astrocytes and microglia, by immunofluorescent co-labeling. Compared to the control group, β-arrestin1 was found significantly decreased in GFAP-positive astrocytes after surgery (Fig. 2D, F). Co-localization of β-arrestin1 with the microglial marker Iba-1 showed no change (Fig. 2E, G). Taken together, orthopedic surgery-induced decrease of β-arrestin1 is mainly found in the reactive astrocytes.

### β-arrestin1 in astrocytes modulates cognitive impairments and astrocytic reactivity in the mouse model for POD

**Fig. 3:**
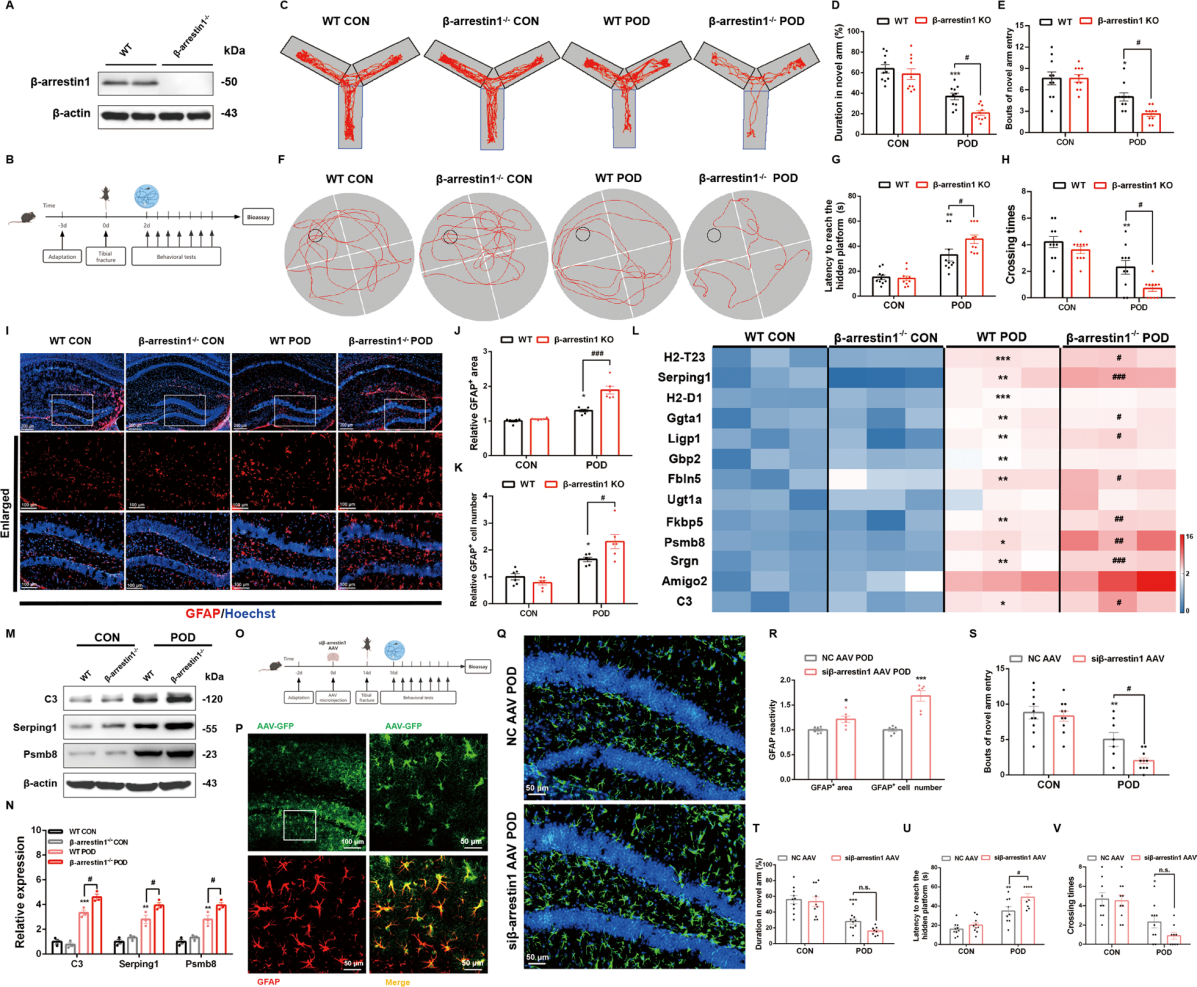
β-arrestin1 in astrocytes modulates cognitive impairments and astrocytic reactivity in the mouse model for POD. **A** β-arrestin1 protein levels in brain lysate of WT and β-arrestin1^−/−^ mice. **B** Experimental protocol and timeline of the POD mouse model. **C** Representative moving track plots (red curve) of mice in the second trial of Y-maze test. Blue box represents the novel arm. **D** Time (%) spent in the novel arm in the Y-maze test. **E** Bouts of novel arm entry in the Y-maze test. **F** Representative moving track plots (red curve) of mice in the probe trial of Morris water maze test. Black circle represents the invisible platform. **G** Latency (s) to reach the hidden platform in the probe test of Morris water maze test. **H** Crossing times in target quadrant in the probe test of Morris water maze test. **I** Representative immunofluorescent staining of GFAP in the hippocampus. **J** Analysis of GFAP-positive cell body area in the hippocampus. **K** Analysis of GFAP-positive cell numbers in the hippocampus. **L** Heatmap of the expression level of the neurotoxic astrocytes-specific transcripts in the hippocampus. **M** Expression of C3, Serping1 and Psmb8 in the hippocampus. **N** Densitometric analysis of C3, Serping1 and Psmb8. **O** Schematic diagram of the mice model with micro-injection of AAV-siβ-arrestin1 into the hippocampus. **P** Immunofluorescent co-localization of GFP and GFAP (red) after the AAV micro-injection. **Q** Representative immunofluorescent staining of GFAP (red) in the hippocampus. **R** Analysis of astrocytic reactivity by GFAP-positive cell body area and GFAP-positive cell numbers in the hippocampus. **S** Bouts of novel arm entry in the Y-maze test. **T** Time (%) spent in the novel arm in the Y-maze test. **U** Latency (s) to reach the hidden platform in the probe test of Morris water maze test. **V** Crossing times in target quadrant in the probe test of Morris water maze test. For C-M except for R, data were analyzed by two-way ANOVA followed by Tukey's multiple comparisons test. ^*^P < 0.05, ^**^P < 0.01 and ^***^P < 0.001 vs. the WT-CON group or NC AAV CON group. ^#^*P* < 0.05, ^##^*P* < 0.01 and ^###^*P* < 0.001 *vs.* the WT-POD mice or NC AAV POD. For R, data are analyzed by unpaired Student’s t-test. ^*^P < 0.05 and ^***^P < 0.001 vs. the NC AAV POD group. *n* = 6 mice per group for immunofluorescent staining. *n* = 3 for western blotting. *n* = 7–10 mice for behavioral tests. Values are presented as means ± SEM

To figure out whether β-arrestin1 plays a role in the pathogenesis of POD, we established orthopedic surgery mouse model using β-arrestin1-knockout mice (Fig. 3A) and then measured the pathological phenotypes including behavioral deficits and brain inflammatory responses as the schematic diagram in Fig. 3B. Y maze test and Morris water maze test were used for cognitive function detection [33]. We found that surgery-treated mice showed significant decrease in the bouts of novel arm entrance and the duration in the novel arm of the Y maze (Fig. 3C–E), implying the decreased learning and memory abilities in surgery-treated mice compared with the control mice. In addition, β-arrestin1 deletion aggravated these impaired abilities of mice (Fig. 3C–E). Results from Morris water maze test showed that the surgery-treated mice took longer time to reach the hidden platform and showed reduced bouts of platform area crossing during hidden-platform acquisition trial compared with those of control mice (Fig. 3F–H). These results demonstrated that β-arrestin1 deletion significantly exacerbated the motor performance deficits and the spatial learning and memory impairments induced by the surgery.

As we found that the decreased expression of β-arrestin1 was shown in reactive astrocytes, we next assessed astroglial activation and astrogliosis in β-arrestin1-deficient POD mice. Compared to control mice, POD mice had numerous activated astrocytes in DG regions, manifested as proliferative morphology of the GFAP-labeled astrocytes (Fig. 3I, J), as well as increased numbers of the reactive astrocytes (Fig. 3I, K). Deletion of β-arrestin1 exacerbated astrocytic reactivity (Fig. 3I–K). We also detected the mRNA levels of the representative neurotoxic astrocytes markers. As shown in Fig. 3L, β-arrestin1 deficiency had no discernable effects on the mRNA levels of these markers under basal conditions, but sharpened their increase in POD mice. We also detected protein levels of representative markers of neurotoxic astrocytes, including C3, Serping1 and Psmb8 and we found that β-arrestin1 deficiency aggravated the increased protein levels in POD mice (Fig. 3M, N). These results implies that β-arrestin1 deletion exacerbates the cognitive impairments of POD mice and the neurotoxic astrogliosis in the hippocampus of this mouse model.

Furthely, we specifically knocked down β-arrestin1 in astrocytes by AAV micro-injection into the hippocampus to investigate whether astrocytic β-arrestin1 was the major mediator in POD pathogenesis. The schematic diagram is shown in Fig. 3O, in which orthopedic surgery mouse model was established after successful micro-injection of AAV into the hippocampus (Fig. 3P). The astrogliosis in POD mice with NC AAV injection was significantly increased after astrocytic β-arrestin1 knockdown, manifested as remarkable proliferative morphology of the GFAP-labeled astrocytes and increased numbers of the reactive astrocytes (Fig. 3Q, R). Similarly, we observed astrocytic β-arrestin1 knockdown further decreased bouts of novel arm entrance of POD mice in Y maze test and took longer time to reach the hidden platform in water maze test (Fig. 3S, U), but showed no significant change in residence time in the novel arm of Y maze and bouts of platform area crossing in water maze (Fig. 3T, V), implying that astrocytic β-arrestin1 knockdown partially aggravated learning and cognitive abilities of the POD mice. These in vivo experiments by static micro-injection confirm, in a great measure, that β-arrestin1 in astrocytes is mainly involved in POD pathogenesis.

### β-arrestin1 deletion aggravates the neurotoxic reactivity of primary astrocytes

**Fig. 4:**
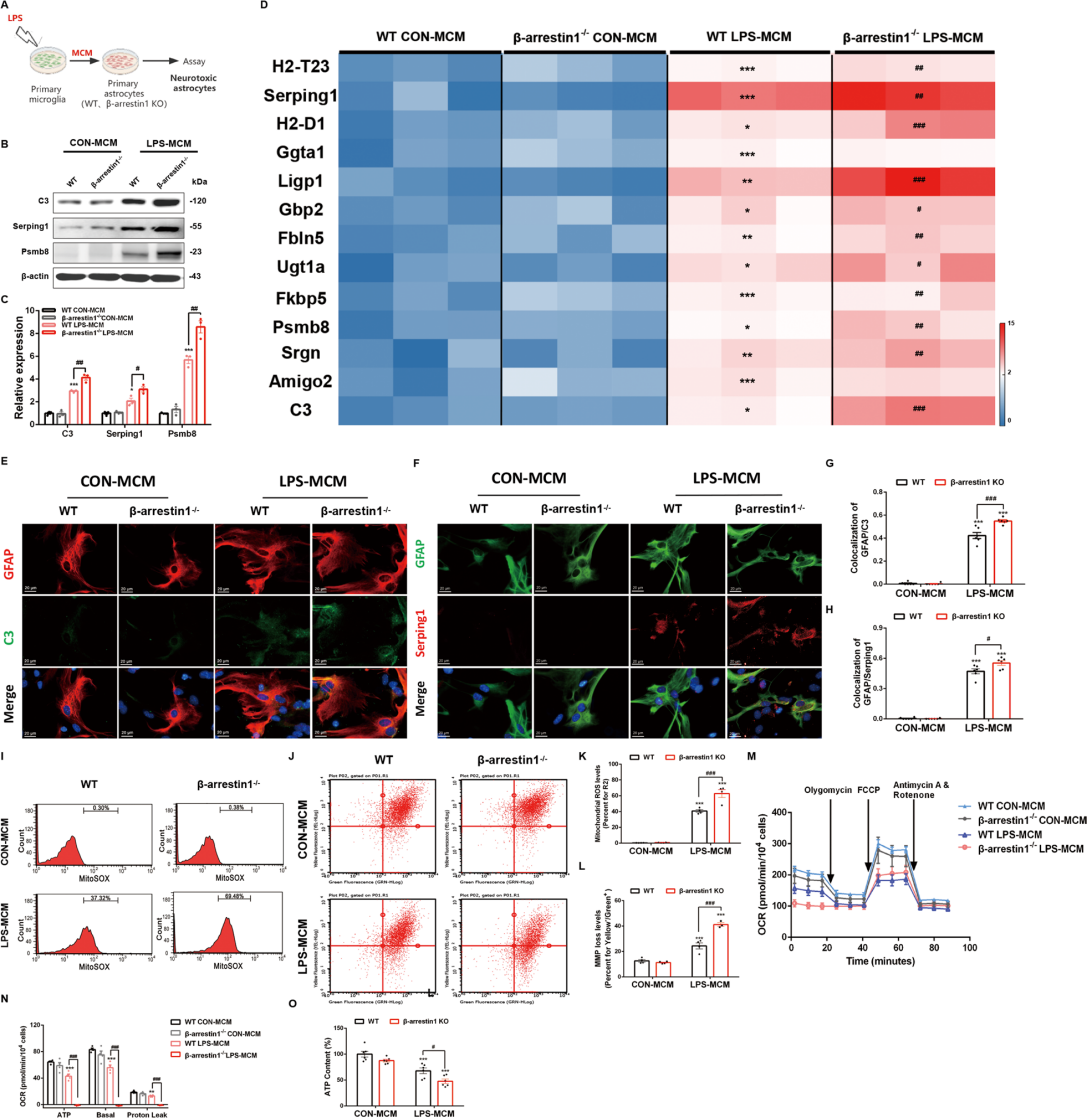
β-arrestin1 deletion aggravates the neurotoxic reactivity of primary astrocytes. **A** Schematic of the experimental design. **B** Expression of C3, Serping1 and Psmb8 in the primary astrocytes. **C** Densitometric analysis of C3, Serping1 and Psmb8. **D** Heat map of A1 astrocytic genes in primary cell cultures. **E** Immunofluorescent staining of C3 (green) and GFAP (red) in primary astrocytes. **F** Immunofluorescent staining of Serping1 (red) and GFAP (green) in primary astrocytes. **G** Relative co-localized signals of the GFAP-positive and C3-positive immunofluorescent particles between groups. **H** Relative co-localized signals of the GFAP-positive and Serping1-positive immunofluorescent particles between groups. **I** Astrocytes were stained with MitoSOX and analyzed by flow cytometry. **J** JC-1 staining in astrocytes were analyzed by flow cytometry. **K** Quantification of the mitochondrial ROS in MitoSOX staining. **L** Quantification of the loss of mitochondrial membrane potential in JC-1 staining measured by flow cytometry. **M** Oxygen consumption rates were evaluated by Seahorse. **N** Quantification of oxygen consumption for ATP production, basal respiration and proton leak. **O** ATP levels in astrocytes. Data were analyzed by two-way ANOVA followed by Tukey's multiple comparisons test. ^*^P < 0.05, ^**^P < 0.01 and ^***^P < 0.001 vs. the WT CON-MCM group. ^#^*P* < 0.05, ^##^*P* < 0.01 and ^###^*P* < 0.001 vs. the WT LPS-MCM group. Values are presented as means ± SEM from at least three independent experiments

To dissect the mechanistic details of β-arrestin1-mediated astrocytic reactivity, we cultured primary astrocytes from WT and β-arrestin1-deficient mice, and induced the astrocytes to the reactive neurotoxic phenotypes. As the neurotoxic astrocytes were originally described as being activated by lipopolysaccharide (LPS)-treated microglia that release IL-1α, TNF, and C1q, we, therefore, stimulated the primary astrocytes with LPS-incubated microglia culture medium (MCM) (Fig. 4A). We first analyzed the C3, Serping1 and Psmb8 levels by immunoblotting analysis (Fig. 4B, C), showing that β-arrestin1 deletion significantly increased the protein levels of the representative markers (Fig. 4B, C). RT-PCR analysis of the markers in primary cultures implied that β-arrestin1-deficient astrocytes are more sensitive to the LPS-MCM stimulation, in which higher mRNA levels of neurotoxic astrocytes markers were shown compared with the WT LPS-MCM group (Fig. 4D). Consistently, the C3-positive signals, as well as Serping1-positive signals in GFAP-labeled astrocytes by immunofluorescent analysis increased visibly in the β-arrestin1-deficient astrocytes with LPS-MCM stimulation (Fig. 4E–H).

As former studies have confirmed that fragmented and dysfunctional mitochondria are involved in glia-mediated inflammatory responses [34], we, therefore, detected whether β-arrestin1 modulated astrocytic reactivity via manipulating mitochondrial dysfunctions. Mitochondrial functions were evaluated by measuring ROS production and the mitochondrial membrane potential. Cells were incubated with MitoSOX Red mitochondrial super-oxide indicator and JC-1 fluorescent dye and then applied to flow cytometric analysis. The results showed that the WT astrocytes treated with LPS-MCM showed higher red fluorescence than the untreated astrocytes, implying increased ROS levels. Meanwhile, β-arrestin1 deletion enhanced the ROS fluorescent signals (Fig. 4I, K). JC-1 assay showed that LPS-MCM induced transformation of red fluorescence to green fluorescence, implying an drastic MMP disruption. β-Arrestin1 deletion drove the fluorescence-transforming trend to a larger extent (Fig. 4J, L). And above all things, functional mitochondria with preserved inner membrane potential show ability to generate ATP [35]. We detected the mitochondrial respiratory functions using Seahorse experiment. We observed a slight decrease, though no significant defects, in oxygen consumption rates of basal respiration, ATP generation and proton leak in β-arrestin1^−/−^ astrocytes (Fig. 4M, N). LPS-MCM prohibited oxygen consumption for basal respiration, ATP generation and proton leak, which were further aggravated after β-arrestin1 deletion (Fig. 4M, N). Similarly, β-arrestin1 deletion under basal condition showed no significant effects on ATP contents, but further reduced the decrease of ATP content caused by LPS-MCM (Fig. 4O). Together, these results show that β-arrestin1 knockout aggravates the neurotoxic phenotype of astrocytes and the mitochondrial dysfunctions.

### β-arrestin1 co-localizes with Drp1 in primary astrocytes

**Fig. 5:**
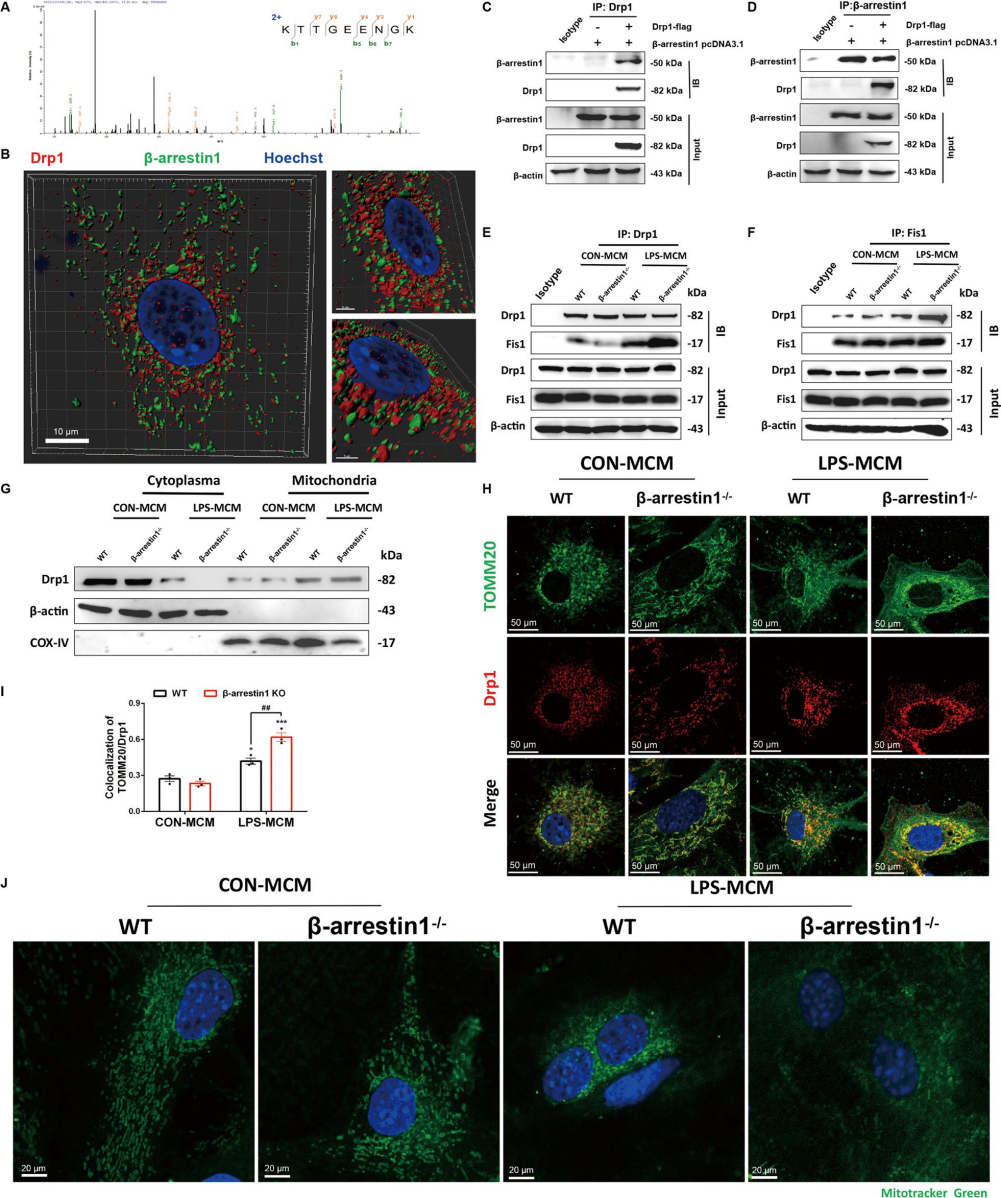
β-arrestin1 co-localizes with Drp1 in primary astrocytes. **A** Identification of Drp1 as a β-arrestin1-associated protein. Mass spectrometry spectrum of a Drp1 tryptic peptide. **B** Flag-tagged Drp1 construct and β-arrestin1 pcDNA3.1 construct were co-transfected in HEK293T cells. Representative immunofluoresent staining of β-arrestin1 (green) and Drp1 (red) processed by Imaris Microscopy Image Analysis software. **C** Flag-tagged Drp1 construct and β-arrestin1 pcDNA3.1 construct were co-transfected in HEK293T cells. Cell lysates were immunoprecipitated with anti-Drp1 antibody and then the samples were analyzed by immunoblotting. **D** Flag-tagged Drp1 construct and β-arrestin1 pcDNA3.1 construct were co-transfected in HEK293T cells. Cell lysates were immunoprecipitated with anti-β-arrestin1 antibody and then the samples were analyzed by immunoblotting. **E** Immunoblotting analysis of Fis1 proteins in cell lysates of primary astrocytes immunoprecipitated with Drp1 antibody. **F** Immunoblotting analysis of Drp1 proteins in cell lysates of primary astrocytes immunoprecipitated with Fis1 antibody. **G** Protein levels of Drp1 in cytoplasmic and mitochondrial parts. **H** Immunofluorescent staining of TOMM20 (green) and Drp1 (red) in primary astrocytes. **I** Relative co-localized signals of the TOMM20-positive and Drp1-positive immunofluorescent particles between groups. **J** Representative images of Mitotracker Green in primary astrocytes under confocal microscope. Data were analyzed by two-way ANOVA followed by Tukey's multiple comparisons test. ^*^P < 0.05 and ^***^P < 0.001 *vs.* the WT CON-MCM group. ^##^*P* < 0.01 vs. the WT LPS-MCM group. Values are presented as means ± SEM from three independent experiments

Accumulating findings demonstrate that β-arrestins can serve as scaffold proteins and function as signal transducers by facilitating interaction of signaling molecules [20–22]. We, therefore, thought deeply of the scaffolding functions of β-arrestin1 in the mitochondrial dysfunctions in reactive astrocytes in the current study. We pulled down β-arrestin1 and then detected β-arrestin1-binding proteins by label-free mass spectrometry, in which we analyzed these proteins related to mitochondrial functions. We found that dynamin-related protein 1 (Drp1), which was highly associated with the neurotoxic astrocytes [34], was identified in the mass spectrometry-identified proteins that bound β-arrestin1 (Fig. 5A). We further detected the co-localization of β-arrestin1 and Drp1 after the plasmids co-transfection within HEK293T cells, we showed that Drp1 interacted with β-arrestin1 and exhibited co-localization by Imaris Image Software (Fig. 5B). Co-immunoprecipitation (CO-IP) assay consistently confirmed the interaction of Drp1 and β-arrestin1 in astrocytes (Fig. 5C, D). As the mitochondrial effector fission protein, Drp1 interacts with mitochondrial fission 1 (Fis1) to facilitate the mitochondrial fission. This process is controlled by the anchorage of Drp1 to Fis1, the receptor in the mitochondrial membrane [36]. We, therefore, assessed the co-localization of Drp1 and Fis1 in the β-arrestin1^−/−^ and WT reactive astrocytes by CO-IP analysis, in which demonstrated that β-arrestin1 deficiency increased the interaction between Drp1 and Fis1 in primary astrocyte cultures (Fig. 5E, F). We then separated the mitochondrial and cytoplasmic parts and detected the Drp1 levels by immunoblotting analysis. As shown in Fig. 5G, mitochondrial Drp1 was significantly up-regulated after treatment of LPS-MCM, whereas β-arrestin1 deletion increased the mitochondrial Drp1 levels, and the cytoplasmic Drp1 was decreased. We also observed the translocation of Drp1 to the mitochondria by immunofluorescently double-staining TOMM20, the mitochondrial marker, and Drp1 (Fig. 5H, I) in the primary astrocytes; and β-arrestin1 deficiency promoted the mitochondrial translocation of Drp1. Furthermore, Mitotracker green, a mitochondrion-selective probe which passively diffuses across the plasma membrane and accumulates in active mitochondria, was used to label the mitochondria and the results in Fig. 5J showed that healthy mitochondria in WT and β-arrestin1^−/−^ control group were circular shapes, while LPS-MCM treatment shaped the mitochondria to be dotted or fragmented patterns. Taken together, β-arrestin1 co-localizes with Drp1 and β-arrestin1 deletion drives the interaction of Drp1 with Fis1 to promote mitochondrial fission.

### β-arrestin1-biased ligand induces the interaction of β-arrestin1 and Drp1 to inhibit mitochondrial fission in vitro

**Fig. 6:**
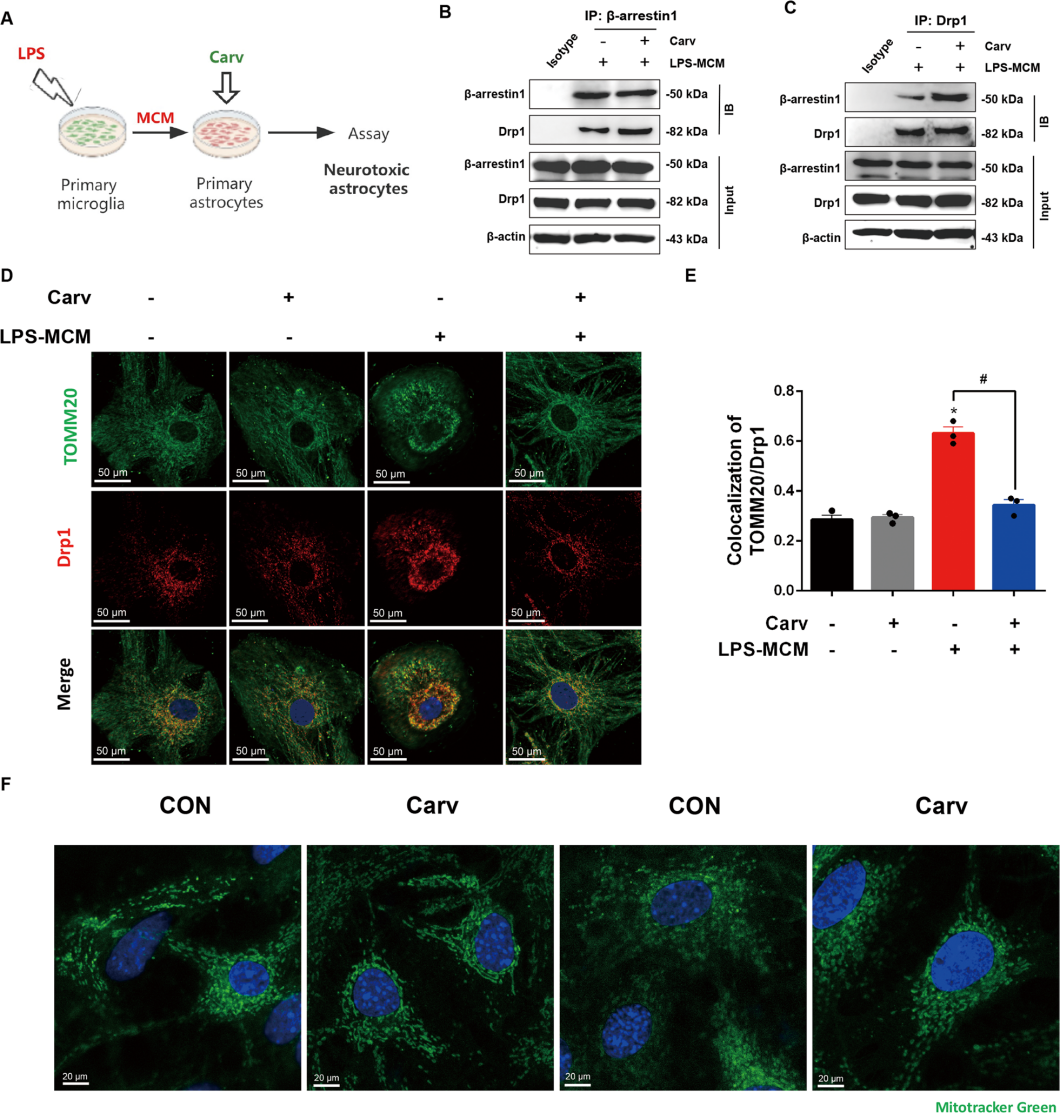
**β**-arrestin1-biased ligand induces the interaction of β-arrestin1 and Drp1 to inhibit mitochondrial fission in vitro. **A** Schematic of the experimental design. **B** Immunoblotting analysis of Drp1 proteins in cell lysates of primary astrocytes immunoprecipitated with β-arrestin1 antibody. **C** Immunoblotting analysis of β-arrestin1 proteins in cell lysates of primary astrocytes immunoprecipitated with Drp1 antibody. **D** Immunofluorescent staining of TOMM20 (green) and Drp1 (red) in primary astrocytes. **E** Relative co-localized signals of the TOMM20-positive and Drp1-positive immunofluorescent particles between groups. **F** Representative images of Mitotracker Green in primary astrocytes under confocal microscope. Data were analyzed by one-way ANOVA followed by Dunnet’s post-hoc test. ^*^P < 0.05 vs. the CON group. ^#^*P* < 0.05 vs. the LPS-MCM group. Values are presented as means ± SEM from three independent experiments

As the preceding data have shown that the regulatory effects of β-arrestin1 on mitochondrial fission by interacting with fission effector protein Drp1, we next asked whether activated β-arrestin1-biased signals would play a role in Drp1-dependent mitochondrial functions. Carvedilol (Carv) is one of the three β-adrenergic receptor (βAR) ligands approved for heart failure, and researches have documented Carv acts on β-arrestin1-biased mechanism to promote cardio-protective actions [37, 38]. We pre-treated the primary astrocytes with Carv and then induced the astrocytes to the reactive states (Fig. 6A). CO-IP analysis was used to detect the combination of Drp1 and β-arrestin1, in which we found that Carv promoted the interaction of these two proteins (Fig. 6B, C). This increased interaction allowed a reversal effect on the translocation of Drp1 to mitochondria induced by the stimuli, shown as a decreased co-localization of Drp1 with mitochondrial marker TOMM20 in Carv-pretreated reactive astrocytes compared with rest astrocytes (Fig. 6D, E). We thus labeled the astrocytic mitochondria with Mitotracker Green to observe their morphology. As shown in Fig. 6F, healthy mitochondria in control group were in circular shapes, while LPS-MCM treatment shaped the mitochondria to be dotted or fragmented patterns. Carv pretreatment protected the astrocytic mitochondria from excessive fragmentation. Taken together, these results demonstrate that Carv promotes the combination of Drp1 and βarr1 to inhibit the Drp1-dependent mitochondrial dysfunctions.

### β-arrestin1-biased ligand Carvedilol recovers the neurotoxic astrocytes reactivity

**Fig. 7:**
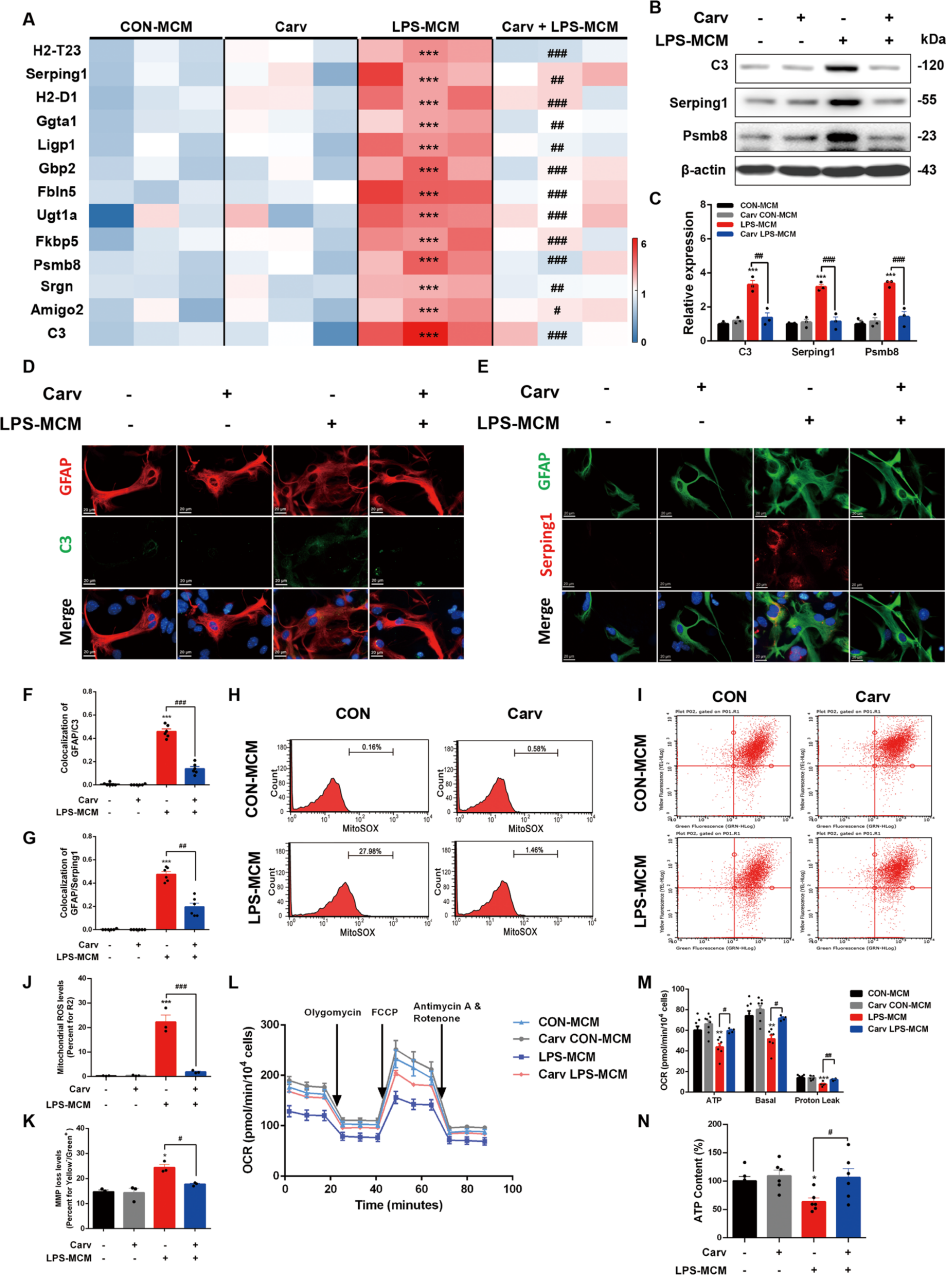
β-arrestin1-biased ligand Carvedilol recovers the neurotoxic astrocytes reactivity. **A** Heat map of the expression levels of the signature genes for neurotoxic astrocytes in primary cell cultures. **B** Expression of C3, Serping1 and Psmb8 in primary cell cultures. **C** Densitometric analysis of C3, Serping1 and Psmb8. **D** Immunofluorescent staining of GFAP (red) and C3 (green) in primary astrocytes. **E** Immunofluorescent staining of GFAP (green) and Serping1 (red) in primary astrocytes. **F** Relative co-localized signals of the GFAP-positive and C3-positive immunofluorescent particles between groups. **G** Relative co-localized signals of the GFAP-positive and Serping1-positive immunofluorescent particles between groups. **H** Astrocytes were stained with MitoSOX and analyzed by flow cytometry. **I** JC-1 staining in astrocytes was analyzed by flow cytometry. **J** Quantification of the mitochondrial ROS in MitoSOX staining. **K** Quantification of the loss of mitochondrial membrane potential in JC-1 staining measured by flow cytometry. **L** Oxygen consumption rates were evaluated by Seahorse. **M** Quantification of oxygen consumption for ATP production, basal respiration and proton leak. **N** ATP levels in astrocytes. Data were analyzed by one-way ANOVA followed by Dunnet’s post-hoc test. ^*^P < 0.05, ^**^P < 0.01 and ^***^P < 0.001 *vs.* the CON group. ^#^*P* < 0.05, ^##^*P* < 0.01 and ^###^*P* < 0.01 vs. the LPS-MCM group. Values are presented as means ± SEM from at least three independent experiments

We next determined the effects of Carv on astrocytes reactivity. We assessed neurotoxic astrocyte markers in primary cultures by RT-PCR and the results confirmed that Carv pretreatment reversed the increased expression of these reactive markers (Fig. 7A). Corroborating these results, immunoblotting analysis revealed decreased levels of the representative markers in the Carv-treated astrocytes with LPS-MCM incubation, compared with the untreated astrocytes with LPS-MCM incubation (Fig. 7B, C). We also examined C3 and Serping1 levels by immunofluorescence and the results showed that C3-positive fluorescent signals, as well as Serping1-positive signals in astrocytes were significantly decreased in astrocytes with Carv pretreatment followed by LPS-MCM stimulation compared with that of untreated astrocytes with LPS-MCM stimulation (Fig. 7D–G). In addition, flow cytometric analysis of MitoSOX fluorescent dye (Fig. 7H, J) and JC-1 assay system (Fig. 7I, K) both showed that Carv pretreatment attenuated the increased ROS fluorescent intensity and transformation of red fluorescence to green fluorescence in the reactive astrocytes, implying a protective effects against mitochondrial malfunctions. We also conducted the mitochondrial oxygen consumption rate by Seahorse experiment. Carv pretreatment showed no effects on the oxygen consumption rates used for basal respiration, ATP generation and proton leak, but recovered the defeats in astrocytes with incubation of LPS-MCM (Fig. 7L, M). ATP assay kit also demonstrated that Carv protected the astrocytes from LPS-MCM-induced ATP decrease (Fig. 7N). Collectively, these data suggest that Carv, the β-arrestin1-biased agonist, exerts protective roles against the reactive signature and mitochondrial abnormalities.

### β-arrestin1-biased ligand Carvedilol protects POD mice from pro-inflammatory phenotypes

**Fig. 8:**
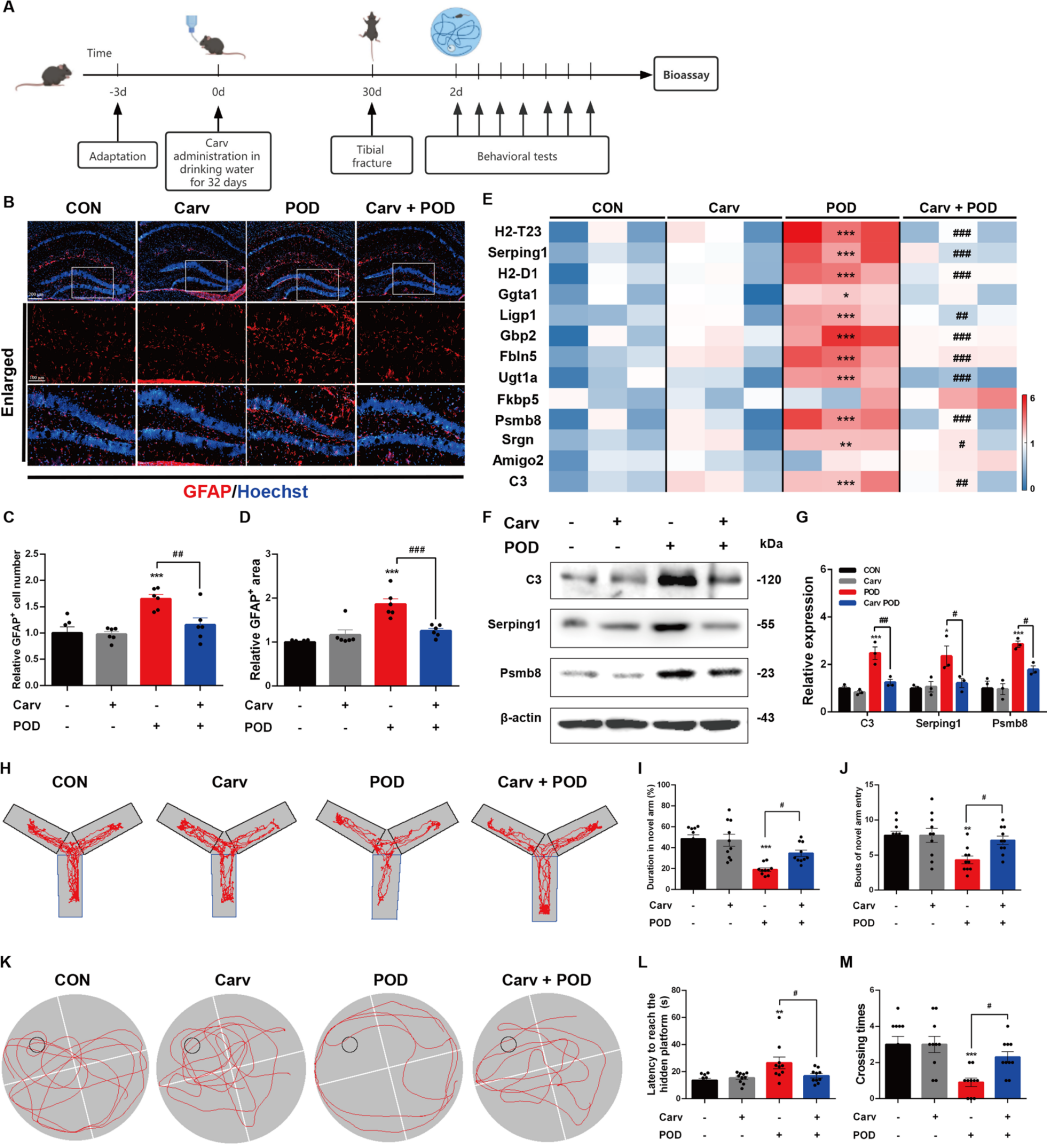
β-arrestin1-biased ligand Carvedilol protects POD mice from pro-inflammatory phenotypes. **A** Experimental protocol and timeline of the mouse model. **B** Representative immunofluorescent staining of GFAP in the hippocampus. **C **Relative GFAP-positive cell body area in the hippocampus. **D** Relative GFAP-positive cell numbers in the hippocampus. **E** Heatmap of the expression level of the A1-specific transcripts in hippocampal samples. **F** Expression of C3, Serping1 and Psmb8 in the hippocampus. **G** Densitometric analysis of C3, Serping1 and Psmb8. **H** Representative moving track plots (red curve) of mice in the second trial of Y-maze test. Blue box represents the novel arm. **I** Time (%) spent in the novel arm in the Y-maze test. **J** Bouts of novel arm entry in the Y-maze test. **K** Representative moving track plots (red curve) of mice in the probe trial of Morris water maze test. Black circle represents the invisible platform. **L** Latency (s) to reach the hidden platform in the probe test of Morris water maze test. **M** Crossing times in target quadrant in the probe test of Morris water maze test. Data were analyzed by one-way ANOVA followed by Dunnet’s post-hoc test. ^*^P < 0.05, ^**^P < 0.01 and ^***^P < 0.001 *vs.* the CON group. ^#^*P* < 0.05, ^##^*P* < 0.01 and ^###^*P* < 0.01 *vs.* the POD group. *n* = 6 mice per group for immunofluorescent staining. *n* = 3 for western blotting. *n* = 10 mice for behavioral tests. Values are presented as means ± SEM

After confirming the inhibitory effects of β-arrestin1-biased ligand on astrocytic reactivity, we next verified its roles in mouse model for POD. The administration strategy of Car is shown in Fig. 8A. By immunofluorescent staining of GFAP to assess astroglial activation and astrogliosis in orthopedic surgery mouse model with Carv administration, we observed that Carv treatment recovered the proliferative morphology GFAP-labeled astrocytes and reduced the numbers of activated astrocytes in DG regions, compared to the POD mice (Fig. 8B–D). By RT-PCR analysis of the hippocampus of the orthopedic surgery mouse model (Fig. 8E), we found that Carv administration significantly contradicted the increased mRNA levels of the markers representing the neurotoxic astrocytic reactivity in POD mice. Analysis of the characteristic markers in protein levels (Fig. 8F, G) revealed consistently reduced expression in the Carv-treated POD mice compared with that of POD mice.

Behavioral tests detecting cognitive functions were also conducted after establishing orthopedic surgery mouse model with Carv administration. It was shown that the surgery-treated mice took longer time to reach the hidden platform and showed reduced bouts of platform area crossing during hidden-platform acquisition trial compared with those of control mice in the Morris water maze test (Fig. 8H–J). Carv administration recovered motor performance deficits and spatial learning and memory impairments in orthopedic surgery mice-induced POD mice (Fig. 8H–J). In Y maze test, Carv abrogated the significant decrease in the bouts of novel arm entrance and the total retention time in the novel arm of the Y maze manifested in surgery-treated mice (Fig. 8K–M), implying its protective roles of Carv in the learning and memory disabilities in surgery-treated mice. Taken together, Carv administration protects POD mice from excessive reactivity of the neurotoxic astrocytes and deficits in cognitive dysfunctions.

### β-arrestin1 deletion negates the inhibitory effects of Carvedilol on neurotoxic astrogliosis and POD progression

**Fig. 9:**
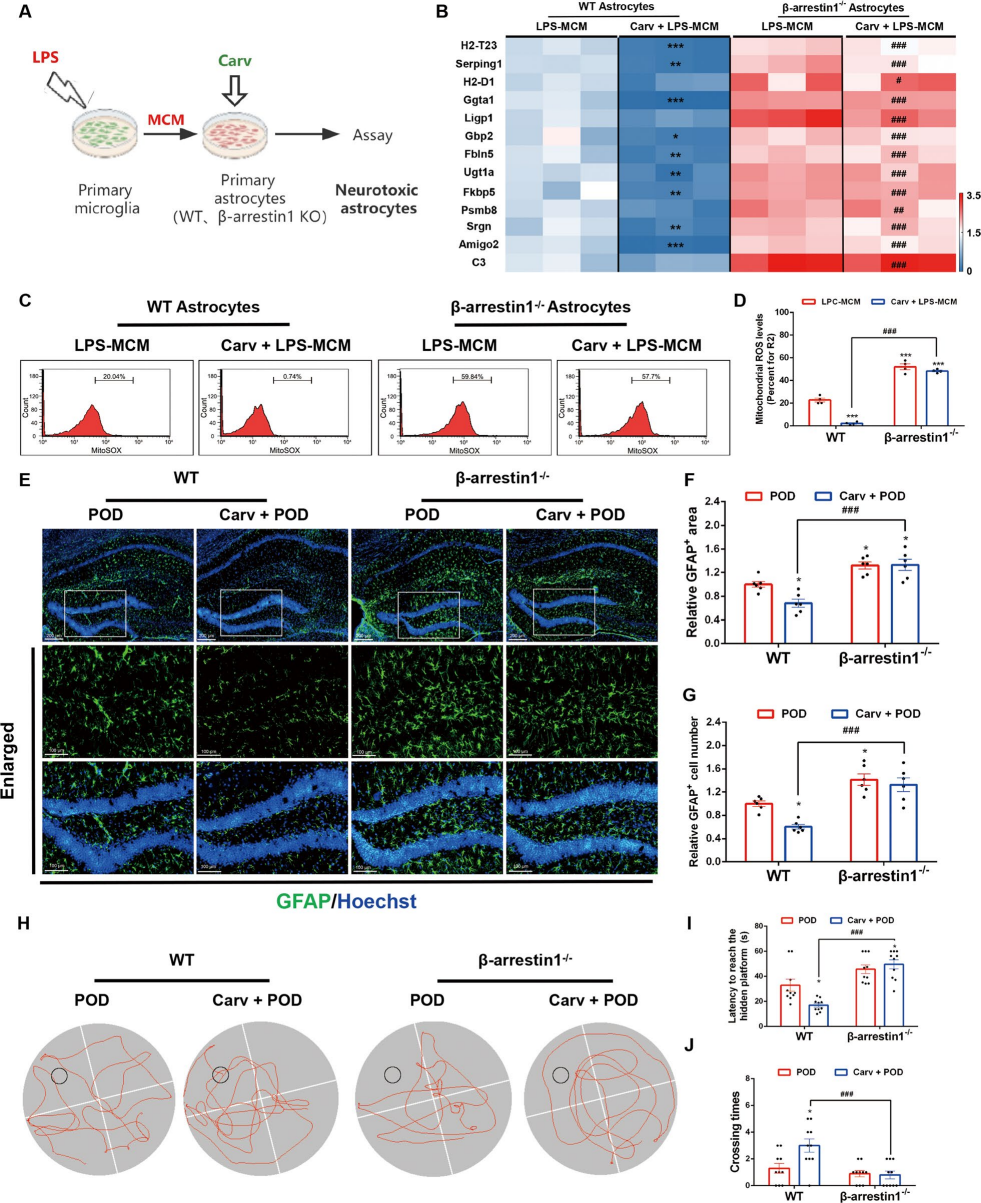
β-arrestin1 deletion negates the inhibitory effects of Carvedilol on neurotoxic astrogliosis and POD progression. **A** Schematic of the experimental design. **B** Heat map of A1 astrocytic genes in primary cell cultures. **C** Astrocytes were stained with MitoSOX and analyzed by flow cytometry. **D** Quantification of the mitochondrial ROS in MitoSOX staining. **E** Representative immunofluorescent staining of GFAP in the hippocampus. **F** Analysis of GFAP-positive cell body area in the hippocampus. **G** Analysis of GFAP-positive cell numbers in the hippocampus. **H** Representative moving track plots (red curve) of mice in the probe trial of Morris water maze test. Black circle represents the invisible platform. **I** Latency (s) to reach the hidden platform in the probe test of Morris water maze test. **J** Crossing times in target quadrant in the probe test of Morris water maze test. Data were analyzed by two-way ANOVA followed by Tukey's multiple comparisons test. ^*^P < 0.05, ^**^P < 0.01 and ^***^P < 0.001 *vs.* the WT POD group. ^#^*P* < 0.05, ^##^*P* < 0.01 and ^###^*P* < 0.01 vs. the WT Carv + POD group. *n* = 6 mice per group for immunofluorescent staining. *n* = 8–10 mice for behavioral tests. Values are presented as means ± SEM

We also supplementarily explored whether Carvedilol’s roles in astrocytic reactivity and POD pathological process are exclusively dependent on β-arrestin1. In vitro studies using β-arrestin1^−/−^ astrocytes to be treated with Carvedilol and then induced into the neurotoxic astrocytes (Fig. 9A) showed that β-arrestin1 deficiency cancelled Carv’s reversal effects on the increased markers of neurotoxic astrocyts by LPS-MCM stimulation (Fig. 9B). As to the mitochondrial malfunctions of neurotoxic astrocytes, β-arrestin1 deletion also abolished the attenuated effects of Carv on excessive ROS generation induced by LPS-MCM (Fig. 9C, D). These results demonstrate in vitro that Carvedilol relies on β-arrestin1 to regulate the astrocytic reactivity.

Furthermore, β-arrestin1^−/−^ mice were used to verify β-arrestin1’s indispensable roles in carvedilol’s neuroprotective effects. In immunofluorescent staining of GFAP showing the astroglial activation and astrogliosis, we observed that Carv treatment recovered the proliferative morphology GFAP-labeled astrocytes and reduced the numbers of activated astrocytes in WT mice, but not β-arrestin1^−/−^ mice (Fig. 9E–G). Behavioral test using water maze test to observe cognitive function of POD mice displayed that β-arrestin1 knockout completely negated the effects of Carv on the cognitive deficits in POD mice. These results demonstrate the indispensable role of β-arrestin1 in Carvedilol’s therapeutic effects on POD progression. Altogether, Carv relies on β-arrestin1 to inhibit neurotoxic astrogliosis and POD progression.

## Discussion

**Fig. 10:**
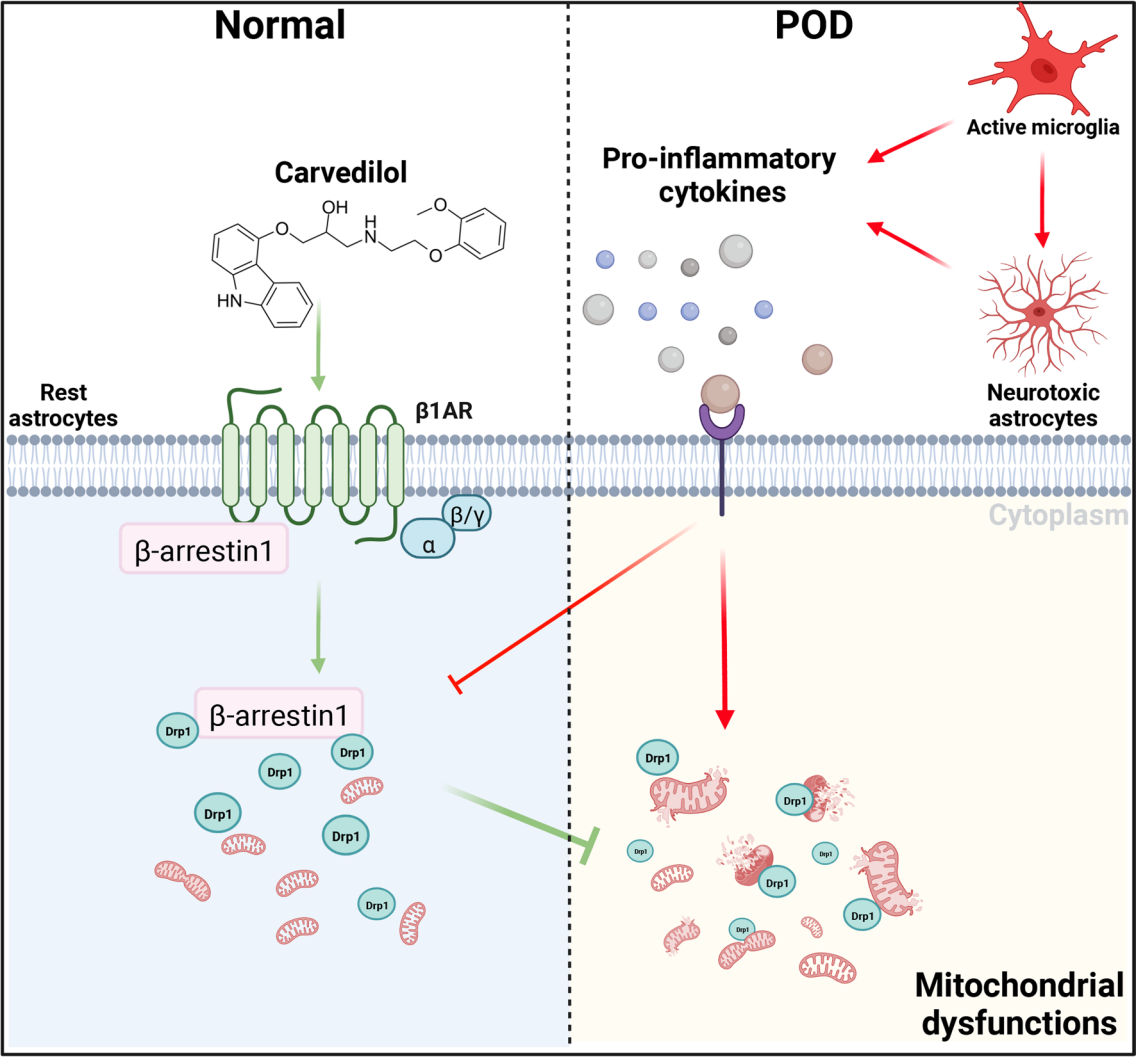
Proposed working model of postoperative delirium triggered by astroglial β-arrestin1 deletion. Decreased β-arrestin1 in postoperative delirium induced astrocytic reactivity to facilitate the pathological progression. β-Arrestin1 deletion triggered the disassociation between with β-arrestin1 and cytoplasmic Drp1 to promote mitochondrial translocation of Drp1 and cause excessive mitochondrial fragmentation and mitochondrial dysfunctions. Activation of β-arrestin1-biased signaling by agonist recovers neurotoxic astrocytic reactivity and phenotypes of postoperative delirium mouse model

In the current study, we showed that orthopedic surgery-induced POD mice exhibited pro-inflammatory phenotypes, as well as excessive neurotoxic astrocyte reactivity in the hippocampus. A gene assay to screen the expression of GPCR family genes showed that β-arrestin1 mRNA levels lowered most remarkably in POD mice. We, therefore, investigated the role of β-arrestin1 in orthopedic surgery-induced postoperative delirium and found that β-arrestin1 deletion showed enhanced cognitive dysfunctions in POD mice and promoted the molecular signature resembling A1-like reactive astrocytes in the mice hippocampus. Further in vitro experiments implied that β-arrestin1-deficient astrocytes were prone to the excessive Drp1-dependent mitochondrial fragmentation and mitochondrial dysfunctions. As β-arrestin1 is reported to be a scaffold protein to transduce intracellular signals by facilitating interaction of signaling molecules, we, therefore, found that β-arrestin1 can interact with cytoplasmic Drp1 to inhibit its translocation to the mitochondrial membrane, which facilitates mitochondrial fragmentation. This process has been proved to be the mechanistic inducer of neurotoxic astrocytes. Further investigations demonstrated that pharmacological manipulation of β-arrestin1-biased signals would prohibit the reactivity of neurotoxic astrocytes and halt pathological progression of postoperative delirium (Fig. 10). We provided direct evidence to reveal that activating β-arrestin1-biased signals recovered the neurotoxic astrocytic reactivity and, therefore, ameliorated cognitive dysfunctions in POD mice.

This study provides insight into the mechanism of astrocytic inflammatory responses and mitochondrial functions regulated by β-arrestin-biased adrenergic receptors. Adrenergic systems are well-studied prototypes for heterotrimeric GPCRs that respond to diffusible hormones and neurotransmitters, which regulate both cognitive function and immune function, and dysregulation of adrenergic tone may potentiate neuroinflammation in neurological diseases [39–41]. Our previous study and other researches found that adrenergic receptors were implicated in the pathogenesis of perioperative neurocognitive disorder [10, 41, 42]. Most notably, we have determined in the current study that β-arrestin1 among the studied GPCR-related genes shows the most significant changes of expression in the brain of mice with postoperative delirium. Although Ccr10 is found to decrease in the POD mice hippocampus in our results, researches have implied that Ccr10 is mainly localized in hippocampal principal cells and apical dendrites of pyramidal neurons [43], implying its potential involvement in neuronal functions rather than astrocytic reactivity. Genetic deletion of β-arrestin1 exacerbated the pathological phenotypes of POD mice. Biased ligands for either G protein-mediated (G protein-biased) or β-arrestin-mediated or β-arrestin-biased signaling could selectively promote beneficial signaling or negate unexpected actions of receptor activation [44]; therefore, they have been intensively investigated recently. β-arrestins have evolved from terminators of G-protein signaling to multifunctional adaptor proteins that form a central node in multiple G-protein-independent signaling pathways. They regulate numerous extracellular signals by communicating with molecules of critical signaling pathways [45]. For example, β-arrestin2 can act as a critical component of the multi-protein GPCR-eNOS signaling complex that promotes eNOS activation and exacerbates the injured liver [46]. β-arrestin1 was a protective mediator in acute pancreatitis via regulation of NF-κB p65 phosphorylation [47]. In the current study, astrocytic β-arrestin1 interacted with Drp1 and, therefore, inhibited its translocation to the mitochondrial membrane to facilitate mitochondrial fission. Under pathological conditions of postoperative delirium, levels of β-arrestin1 decreased, which released more Drp1 to interact with outer mitochondrial membrane protein Fis1, promoted mitochondrial dysfunctions. Furthermore, we speculated that pharmacological activation of β-arrestin1-biased signals would rescued the excessive mitochondrial fission in the reactive astrocytes of POD mice hippocampus. Carvedilol is an anti-hypertensive drug, which is reported as cardio-protective agent via β-arrestin1-mediated β1-adrenoreceptor binding without activating G-proteins, providing an additional mechanism for its clinical efficacy [37]. It also improves age-related cognitive impairment and has been undergoing phase IV used in clinical trials of AD [48]. Here in our study, Carvedilol induced the β-arrestin1-biased signaling and promoted combination of β-arrestin1 and Drp1 to inhibit mitochondrial dysfunctions of neurotoxic astrocytes. This effects underlay its protective roles against the astrocytic inflammatory responses and cognitive impairments of POD mice.

Aseptic surgical trauma triggers acute inflammation by inducing inflammatory cytokines and damage-associated molecular patterns (DAMPs). This inflammatory milieu contributes to the recruitment of immune cells at the site of injury, but also affects functions in other organs, including the brain [9]. Previous clinical investigations demonstrated that in patients who suffered from postoperative delirium or had multiple traumas, there is early C3 activation. C3, as a central component of complement system exerts indispensable roles in the regulation of immune responses and inflammation [16, 49]. In a murine model of orthopedic surgery, C3 was up-regulated in hippocampal astrocytes [16]. Beyond the simplistic view of supporting elements to neurons, astrocytes play essential roles including inflammatory responses and phagocytic activities [50]. Studies have verified a newly identified astrocyte sub-population triggered by the LPS-activated microglia, termed A1 astrocytes [32, 34]. This type of reactive astrocytes, abundant in normal aging and various neurological diseases, have lost most normal astrocytic functions but gain a new neurotoxic functions [51]. As C3 is the characteristic marker of the neurotoxic astrocytes [32], which were altered in mice with postoperative delirium in our study, we, therefore, predicted that postoperative delirium induced astrocytes to be a neurotoxic phenotype. The excessive pro-inflammatory cytokines and decreased anti-inflammatory cytokines and neurotrophic factors observed in the POD mice brain in the current study consistently provide an optimal micro-environment for the neurotoxic astrocytes to function properly [32]. It has been pointed out that multiple molecular and functional parameters are necessary to define reactive astrocytes, rather than binary divisions of reactive astrocytes into or A1-vs-A2 depending on astrocytic transcriptome [52], we conducted multidimensional investigations, including detecting the specific markers and identifying dysfunctional characteristics to reveal the pathological phenotypes of astrocytes during POD progression in the current study.

The dynamic properties of mitochondria include their fusion, fission, transport and degradation; and all of them are critical for their optimal functions [35]. The interplay of fusion and fission confers widespread benefits on mitochondria, including efficient transport, increased homogenization of the mitochondrial population, and efficient oxidative phosphorylation [35]. Mediated by Drp1, mitochondrial fission should be strictly controlled, because excessive mitochondrial fragmentation often involves in the pathogenesis of neurological diseases [36, 53]. Dysfunctional mitochondria released from neurotoxic astrocytes occurs in a Drp1-Fis1-specific manner and suppression of this Drp1-Fis1-dependent process impedes neuronal degeneration [34]. We found in the current study that Drp1 can interact with β-arrestin1 to inhibit its translocation to the mitochondrial membrane and, therefore, prevents Drp1-Fis1-dependent mitochondrial fission. POD-induced decrease of β-arrestin1 may release the cytoplasmic Drp1 to perform mitochondrial fission and, therefore, led to mitochondrial dysfunctions. On the contrary, activation of β-arrestin1 signals facilitated the combination between Drp1 and β-arrestin1 to protect the excessive mitochondrial fission. Although we observed interaction of β-arrestin1 and Drp1, we deduced that this interaction mainly affected the abnormal function of cells under pathological conditions, as no significant mitochondrial malfunctions were observed under basal conditions of β-arrestin1 deletion or supplementation of β-arrestin1-biased agonist. We, therefore, emphasized the cardinal effects of this combination on mitochondrial dynamics under neuroinflammatory stress, which might conceal the compensatory effects under normal physiological state.

Despite the interesting findings presented here, it is worth noting the potential limitations of the present study. First, we used healthy mice as the controls in our in vivo studies, instead of delirium-resistant surgery mice for the following two reasons. For one reason, surgery with anesthesia did not uniformly induce behavioral defects in both water maze test and Y-maze test due to symptomatic differences in mice. And also there is no precise and widely-recognized behavioral scoring rules that define delirium and non-delirium mice for the orthopedic surgery-induced mouse model like the clinical Delirium Screening Scale does. Therefore, there are some difficulties in clearly distinguishing the delirium-susceptible mice and the delirium-resistant mice undergoing orthopedic surgery with general anesthesia. For another more important reason, general anesthesia surgery can be a sub-threshold stimulation in individuals, which induces stable and precise pathological changes in the CNS including neuroinflammation and hippocampal atrophy without delirium behaviors. That is to say, the pathological process of POD in mice with non-delirium orthopedic surgery may be already underway. Together, healthy mice was used as the preferable controls to study the molecular pathology of POD in our research. Secondly, we used LPS-MCM in our in vitro studies, which could only replicate the in vivo POD environment to some extent. The process of microglia activation (by LPS) mimicks the pro-inflammatory micro-environment of POD for neurotoxic astrogliosis, but this is not the POD-specific condition.

## Conclusions

In summary, our findings demonstrate for the first time that β-arrestin1 is involved in the progression of postoperative delirium. The underlying mechanism is mediated by Drp1-dependent mitochondrial fission and mitochondrial dysfunctions in reactive astrocytes. Activation of β-arrestin1 biased signals provide novel insights into POD therapeutics. This study extends our understanding of the pathogenesis of postoperative delirium and may aid in the development of drugs for the treatment of POD.

## Data Availability

The raw data that support the findings of this study are available from the corresponding author, upon reasonable request.

## Abbreviations

AD: Alzheimer’s disease
β-AR: β-adrenergic receptor
BDNF: Brain derived neurotrophic factor
C3/C3R: Complement component 3/complement component 3 receptor
Carv: β-arrestin1-biased agonist Carvedilol
CNS: Central nervous system
DAMPs: Damage-associated molecular patterns
DG: Dentate gyrus
Drp1: Dynamin-related protein 1
ERK: Extracellular signal-regulated kinase
TNF-α: Tumor necrosis factor-α
GFAP: Glial fibrillary acidic protein
GPCRs: Guanine nucleotide-binding protein (G protein)-coupled receptors
HIF-1α: Hypoxia-inducible factor 1α
Iba-1: Ionized calcium-binding adapter molecule 1
IL-1β: Interleukin-1β
PD: Parkinson's disease
POD: Postoperative delirium
WT: Wild type
